# Natural Compound ZINC12899676 Reduces Porcine Epidemic Diarrhea Virus Replication by Inhibiting the Viral NTPase Activity

**DOI:** 10.3389/fphar.2022.879733

**Published:** 2022-05-04

**Authors:** Pengcheng Wang, Xianwei Wang, Xing Liu, Meng Sun, Xiao Liang, Juan Bai, Ping Jiang

**Affiliations:** ^1^ Key Laboratory of Animal Disease Diagnostics and Immunology, Ministry of Agriculture, MOE International Joint Collaborative Research Laboratory for Animal Health and Food Safety, College of Veterinary Medicine, Nanjing Agricultural University, Nanjing, China; ^2^ Jiangsu Co-Innovation Center for Prevention and Control of Important Animal Infectious Diseases and Zoonoses, Yangzhou, China

**Keywords:** ZINC12899676, PEDV replication, virtual screening, nsp13, NTPase, deep learning

## Abstract

Porcine epidemic diarrhea virus (PEDV) is an alphacoronavirus (α-CoV) that causes high mortality in suckling piglets, leading to severe economic losses worldwide. No effective vaccine or commercial antiviral drug is readily available. Several replicative enzymes are responsible for coronavirus replication. In this study, the potential candidates targeting replicative enzymes (PLP2, 3CLpro, RdRp, NTPase, and NendoU) were screened from 187,119 compounds in ZINC natural products library, and seven compounds had high binding potential to NTPase and showed drug-like property. Among them, ZINC12899676 was identified to significantly inhibit the NTPase activity of PEDV by targeting its active pocket and causing its conformational change, and ZINC12899676 significantly inhibited PEDV replication in IPEC-J2 cells. It first demonstrated that ZINC12899676 inhibits PEDV replication by targeting NTPase, and then, NTPase may serve as a novel target for anti-PEDV.

## Introduction

Porcine epidemic diarrhea virus (PEDV), a member of the genera *Alphacoronavirus* in the Coronavirinae subfamily, widely spreads and causes lethal diarrhea in suckling piglets across the world (Jung and Saif, 2015). Due to multiple transmission routes and the case of wild boars harboring PEDV, the commercial swine industry faces greater challenges (Lee et al., 2016; Bevins et al., 2018). Co-infections of PEDV with other viruses complicate disease control and prevention (Chen et al., 2018; Guo et al., 2020; Zhang H et al., 2021). Unfortunately, there is still a lack of effective vaccines against PEDV (Kweon et al., 1999; Lee, 2015; Lin et al., 2016). Antiviral drugs may be a promising pathway to fight against viral infections. For example, acyclovir is approved for the treatment of primary and recurrent genital herpes simplex virus (HSV) infection (Razonable, 2011). Oseltamivir is a licensed medication that specifically targets the neuraminidase protein common to influenza A and B viruses (Long et al., 2018). The antiviral drug molnupiravir for the treatment of mild-to-moderate COVID-19 in adults with at least one risk factor for severe illness has been authorized in UK since November 2021 (Mahase, 2021a). It has been reported that certain small molecules inhibit PEDV proliferation, but commercial drugs have not been developed yet (Wang et al., 2020; Huan et al., 2021; Zhang Y et al., 2021).

Several replicative enzymes responsible for coronavirus replication may be the potential target of antiviral treatment. Papain-like protease (PLpro) plays an important role in virus maturation, dysregulation of host inflammation, and antiviral immune responses (Shin et al., 2020). It can be inhibited by GRL0617, a benzamide resulting from the formal condensation of the carboxy group of 5-amino-2-methylbenzoic acid with the amino group of (1R)-1-(naphthalen-1-yl)ethan-1-amine, which further showed a clear inhibition of SARS-CoV-2 replication *in vitro* (Fu et al., 2021). Paxlovid is reported in phase III trials for the treatment of COVID-19 by targeting 3C protease (3CLpro), which is essential for processing and maturation of the viral polyprotein (Mahase, 2021b; Zhao et al., 2021). The approved antiviral drug molnupiravir increases the frequency of viral RNA mutations and impairs SARS-CoV-2 replication by disturbing the function of RNA-dependent RNA polymerase (RdRp) (Kabinger et al., 2021). Researchers identified and validated a compound (a bismuth salt) that specifically blocks nucleoside triphosphate hydrolase (NTPase) and RNA helicase activities of SARS-CoV-2 nsp13 (Shu et al., 2020). It is reported that coronavirus uridylate-specific endoribonuclease (EndoU) ensures efficient viral replication (Zhao et al., 2020), and compound NSC95397, also known as 2,3-bis(2-hydroxyethylthio)-1,4-naphthalenedione, was screened as an inhibitor of EndoU (Canal et al., 2021). However, the discovery and screening of small compounds targeting PEDV replicative enzymes have not been systematically conducted up to now.

High-throughput screening has been used for screening of chemical libraries to discover lead compounds for target (Sundberg, 2000). The interactions between compound libraries and target can be evaluated by virtual screening in a rapid and low-consumption manner (Ma et al., 2013). Several antiviral drugs against SARS-CoV-2, Ebola virus, Zika virus, and so on have been found through virtual screening (Capuzzi et al., 2018; Santos et al., 2020; Jang et al., 2021).

To discover the antiviral compound against PEDV, potential PEDV replicative enzyme inhibitors have been identified by virtual screening of 187,119 compounds contained in the ZINC natural products database. It was observed that NTPase had the most hits among the targets. Among the chosen seven compounds, ZINC12899676 was identified to significantly inhibit the NTPase activity. Furthermore, ZINC12899676 was confirmed to significantly inhibit PEDV replication *in vitro*. These findings offer novel and promising therapeutic possibilities for fighting infections caused by PEDV.

## Materials and Methods

### Hardware, Software, and Web Service

Virtual screening and molecular dynamics (MD) simulation studies were performed using a 12-core Intel Xeon Platinum 8269CY processor rented from the Aliyun platform. AutoDock Vina (Trott and Olson, 2010) and LeDock (Wang Z et al., 2016) were used in two-round virtual screening. MD simulations were conducted using the GROMACS package and the GROMOS96 43A1 force field, according to the previous description. LigPlot+ is a program for automatic generation of 2D ligand–protein interaction diagrams. Electrostatic potential surfaces were analyzed by Gaussian and visualized by Multiwfn. The webtool ADMETlab2.0 was used to evaluate the drug-like properties of the compounds (Xiong et al., 2021).

### 3D Model Generation and Verification of Targets

Unknown structure targets were modeled by using three methods: I-TASSER, designed for protein structure modeling by iterative threading assembly simulations (Yang J. et al., 2015; Roy et al., 2010; Zhang, 2008); SWISS-MODEL, dedicated to protein structure homology modeling (Waterhouse et al., 2018); and trRosetta, built for fast and accurate protein structure prediction by deep neural network and homology modeling (Du et al., 2021). C-score of I-TASSER is typically in the range of [−5, 2], where a higher C-score signifies a model with a high confidence and vice versa. A TM-score of I-TASSER greater than 0.5 indicates a model of correct topology. GMQE gives an overall model quality measurement to the SWISS-MODEL between 0 and 1, with higher numbers indicating higher expected quality. A TM-score of trRosetta higher than 0.5 usually indicates a model with correctly predicted topology. Quality assessment of the predicted 3D model of targets was performed using VERIFY 3D (Luthy et al., 1992), PROCHECK (Laskowski et al., 1996), WHATCHECK (Hooft et al., 1996), ERRAT (Colovos and Yeates, 1993), and PROVE (Pontius et al., 1996) integration in SAVES v6.0.

### Choice of the Small Compound Library

Natural products library of ZINC15 (Sterling and Irwin, 2015), which is a free database of commercially available compounds provided by the Irwin and Shoichet Laboratories in the Department of Pharmaceutical Chemistry at the University of California comprising 187,119 compounds, was selected for virtual screening. The structure files of ligands were transformed into PDBQT and mol2 files for AutoDock Vina and LeDock by Raccoon and Open Babel software.

### Prokaryotic Expression and Purification of PEDV nsp13

The full-length cDNA of PEDV nsp13 was amplified *via* PCR using the primers, 5′-CGC​GAA​TTC​TCT​GCA​GGG​CTT​TGT​GTT​GTT-3′ and 5′-GCG​CTC​GAG​CTG​CAA​ATC​AGA​CAA​TTT​AAG-3′, and then, it was inserted into a pET-28a vector at EcoRI/XhoI restriction sites. The pET-28a-nsp13 was transformed into the *E. coli* strain BL21, and the cells were cultured at 37°C in the LB medium. When optical density at 600 nm (OD600) reached 0.8, the culture was cooled to 18°C and supplemented with 0.2 mM IPTG. The cells were harvested after incubation at 18°C for 18 h, resuspended in PBS, and then disrupted by ultrasonication. The supernatant was filtered and loaded on Ni-sepharose (GE Healthcare, United States). Finally, the His-tagged protein was eluted using a linear gradient between binding buffer and elution buffer A (20 mM Tris, pH 7.4, 500 mM NaCl, and 250 mM imidazole). A low concentration of imidazole (50 mM) was used to wash impurities, and a high concentration of imidazole (250 mM) was used to elute targeted protein. The target protein was condensed and desalinated using an Amicon Ultra-4 centrifugal filter unit (50 kDa, GE Healthcare, United States). The proteins were analyzed by SDS-PAGE. All the purification procedures were performed at 4°C to avoid unexpected degradation.

### ATPase Activity Assay

The Kinase-Glo Plus Luminescent Kinase Assay kit (Promega, United States) was used to detect the ATPase activity of PEDV nsp13. In brief, the purified recombinant proteins in reaction buffer (40 mM Tris-HCl at pH 7.5, 50 mM NaCl, 2 mM Mg^2+^, 80 μM ATP, and 0.2 μM NTPase) were added to a 96-well black plate in the presence of compounds or DMSO to a total volume of 50 μl. Apyrase from potatoes (Sigma-Aldrich, United States) was used as a positive control enzyme. PEDV N protein was used as a negative control enzyme. The plate was then incubated in a 37°C incubator for 20 min. At the end of the reaction, 50 μl of Kinase-Glo reagent was added to the reaction mixture. After mixing and incubating at room temperature for 2 min, luminescence of each well was measured using a PerkinElmer EnSpire Multimode Plate Reader.

### MM-PBSA

The molecular mechanics Poisson–Boltzmann Surface Area (MM-PBSA) approach has been used to estimate the free energy of the binding of small ligands to biological macromolecules. The gmx_mmpbsa shell (https://jerkwin.github.io/gmxtool) together with the APBS program was used to calculate ligand-binding affinities and the corresponding energy decomposition term based on molecular dynamics trajectory (Du et al., 2022). Free binding energy lower than −57 kJ/mol was regarded as very strong affinity.

### FEL

The free energy landscape (FEL) is a representation of possible conformations taken by a protein or a ligand–protein complex in molecular dynamics simulation along with Gibbs free energy. Both 2D and 3D graphs of the FEL were plotted using Rg, RMSD, and Gibbs free energy, calculated by using the gmx sham tool.

### Cells, Virus, and Reagents

Vero cells and IPEC-J2 cells were maintained in Dulbecco’s modified Eagle’s medium (DMEM) (Gibco, United States) with 10% fetal bovine serum (Lonsera, Uruguay), penicillin (250 U/ml), and streptomycin (250 µg/ml). The cells were incubated at 37°C in a humidified incubator with 5% CO_2_. The PEDV MS (GenBank accession no. MT683617) strain was maintained in our laboratory and passaged in Vero cells with 6 μg/ml trypsin. Small compounds with purity >90% were used for *in vitro* experiments (Molport, United States).

### Viral Inhibition Assay *In Vitro*

Vero and IPEC-J2 cells were seeded in 24-well plates at 5 × 10^5^ cells per well. Due to the difference in sensitivity to PEDV, different MOIs of PEDV have been used for Vero and IPEC-J2 cells (Altawaty et al., 2018; Zhang et al., 2018). When approximately 90% confluent, the cells were treated with a compound of different concentrations or DMSO (0.1% v/v) for 1 h and then infected with PEDV (0.01 MOI for Vero and 1 MOI for IPEC-J2) or mock infected for 1 h. The cells were then washed with PBS, and then, the culture medium containing the compound was added back to each well. At 16 h post-infection (hpi), samples were collected to detect the virus quantity. Cell viability was tested using an enhanced Cell Counting Kit-8 (CCK-8) (Beyotime, China), following the manufacturer’s instructions.

### Virus Titration

Vero cells grown in 96-well plates were infected with 10-fold serial dilutions of PEDV samples in four replicates. After 1 h at 37°C, the culture medium was replaced with fresh DMEM. The plates were incubated for 48–72 h at 37°C. Virus titers are expressed as TCID_50_, calculated using the Reed–Muench method.

### RNA Extraction and Quantitative Real-Time PCR

Total RNA was extracted from the IPEC-J2 cells using a Total RNA kit (Omega Bio-tek, United States) and reverse transcribed into cDNA using HiScript qRT SuperMix (Vazyme, China), following the manufacturer’s instructions. The detection of mRNA levels of PEDV was performed, as described previously (Wang et al., 2020). Quantitative RT-PCR was performed using the AceQ^®^ qPCR SYBR^®^ Green Master Mix (Vazyme, China). Each reaction was performed in triplicate, and results are expressed as mean ± standard deviation (SD).

### Western Blot Assay

The cells were lysed with 100 μlof RIPA lysis buffer (Beyotime, China) on ice for 15 min, then resolved by SDS-PAGE, and transferred to a nitrocellulose membrane. After transfer, the membrane was incubated in blocking buffer (5% non-fat milk in PBST w/v) for 2 h at room temperature, washed three times with PBST, and then probed with the following antibodies: anti-PEDV N-protein (1:1000) prepared in our laboratory; anti-GAPDH (60004-1-Ig, 1:5000; Proteintech Group, United States); and anti-His (1:2000; AF5060, Beyotime, China) for 2 h at room temperature; then, it was washed three times with PBST. The membranes were incubated with HRP-conjugated goat anti-mouse IgG (H−L) secondary antibodies (1:1000; A0216, A0208, Beyotime, China). Bound proteins were exposed with an ECL Kit (Tanon, China).

### Construction of the Deep Learning Network

A simple multilayer perceptron (MLP) neural network containing three hidden layers was built to predict the binding energy of ultra-large compound library in-stock. The nodes in the network used a non-linear rectified linear unit (ReLU) function as the activation function. The model was trained using the adaptive moment estimation (Adam) optimizer, which estimates the adaptive learning rate for all parameters involved in the training of gradients. The relationship of the simplified molecular-input line-entry system (SMILES) string and binding energy data from virtual screening was used to train a regression model. We divided the data set into the training set, test set, and validation set with the ratio of 7:2:1. The mean squared error (MSE) was utilized as the loss function and performance metrics:

$$MSE=\frac{1}{N}\sum_{t=1}^{N}{\left(observed_{t}-predicted_{t}\right)}^{2}.$$

The hyperparameters, including the learning rate, neuron number in each layer, batch size, and epoch, were tuned.

### Statistical Analysis

Statistical analysis was performed by GraphPad Prism 8 software. Results were expressed as mean ± SD. Differences between groups were examined for statistical significance using one-way analysis of variance (ANOVA). The asterisks in the figures indicated significant differences (* = *p* < 0.05; ** = *p* < 0.01; *** = *p* < 0.001; ns, not significant).

## Results

### Virtual Screening

PEDV replicative enzymes were modeled by I-TASSER, SWISS-MODEL, and trRosetta webtool. All but the 3D structure of nsp12 modeled by trRosetta were credible according to the self-standard (Table 1A). The structures were then subjected to validation by using structure validation tools such as VERIFY 3D, PROCHECK, ERRAT, and PROVE (Table 1B). We selected the structure of nsp12 modeled by using SWISS-MODEL and structures of nsp13 and nsp15 modeled by trRosetta (Table 2). Key amino acid residues were set as pocket for virtual screening.

**TABLE 1A:** Targets were modeled by using three methods.

Predicted protein	I-TASSER		SWISS-MODEL		trRosetta
	C-score	TM-score	GMQE	Seq similarity	TM-score
nsp3	1.54	0.93 ± 0.06	0.99	1.00	0.849
nsp5	1.77	0.96 ± 0.05	0.96	1.00	0.916
nsp12	1.53	0.93 ± 0.06	0.83	0.49	0.389
nsp13	1.59	0.94 ± 0.05	0.81	0.49	0.609
nsp15	1.80	0.97 ± 0.05	0.88	0.52	0.889

**TABLE 1B:** Quality assessment of the predicted 3D model of targets.

	Prediction method	VERIFY 3D		PROCHECK			ERRAT	PROVE	
				Errors	Warning	Pass			
	I-TASSER	Pass	96.60%	6	1	1	97.7169	Warn	5.0%
nsp3	SWISS-MODEL	Pass	96.17%	0	4	4	97.235	Warn	2.9%
	trRosetta	Pass	97.45%	0	5	3	93.2432	Fail	5.9%
	I-TASSER	Pass	98.01%	5	1	2	98.2993	Warn	3.6%
nsp5	SWISS-MODEL	Pass	98.84%	2	5	2	94.6809	Warn	2.9%
	trRosetta	Pass	98.34%	1	4	3	92.1429	Warn	3.1%
	I-TASSER	Pass	87.12%	6	1	1	94.7195	Fail	6.1%
nsp12	SWISS-MODEL	Pass	93.48%	6	1	2	87.5449	Fail	7.6%
	trRosetta	—	—	—	—	—	—	—	—
nsp13	I-TASSER	Pass	98.66%	6	1	1	89.4378	Fail	5.3%
	SWISS-MODEL	Pass	97.30%	1	5	2	96.6252	Warn	3.4%
	trRosetta	Pass	93.47%	5	1	3	90.3282	Warn	4.8%
nsp15	I-TASSER	Pass	91.15%	4	3	1	94.5619	Warn	4.6%
	SWISS-MODEL	Pass	94.71%	2	5	2	94.8825	Warn	3.4%
	trRosetta	Pass	95.87%	1	4	3	95.9502	Warn	4.4%

**TABLE 2:** Summary of selected replicative enzymes and 3D structures.

Protein name	Alternative name	Target site	Structure used
nsp3	PLP2	C1729 H1888 D1901 W1730	Chain O extracted from 7MC9
nsp5	3CLpro	H41 C144	4XFQ
nsp12	RdRp	G608 W609 D610 Y611 L750 S751 D752 D753 K790 C791 W792 E803 F804 C805 S806	Structure modeled by SWISS-MODEL
nsp13	NTP	K289 S290 D375 E376 Q405 R568	Structure modeled by trRosetta
nsp15	NendoU	H226 H241 K282	Structure modeled by trRosetta

**FIGURE 1:**
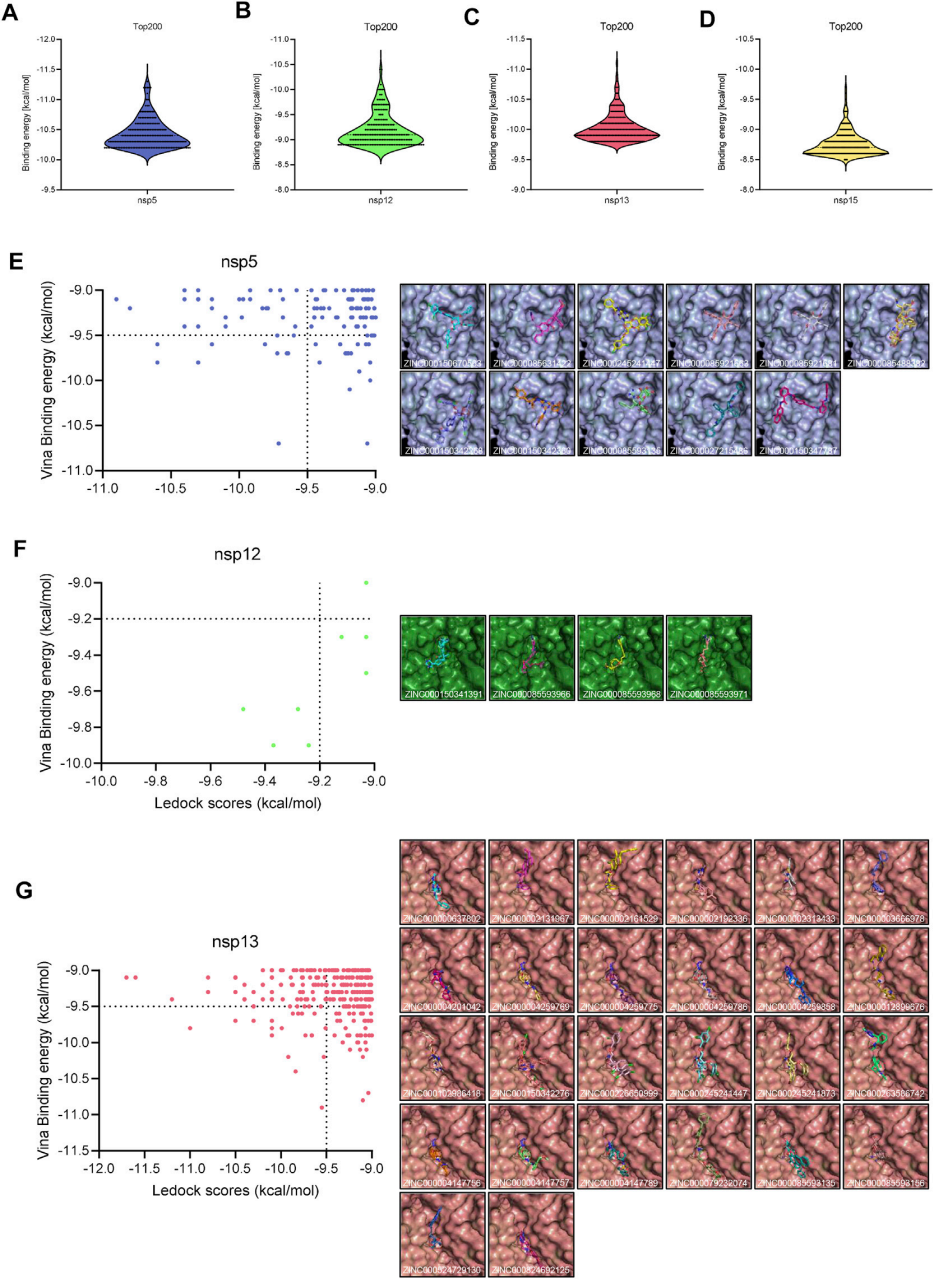
Virtual screening. **(A**–**C)** Distribution of the docking scores of the top 200 virtual screening hits by AutoDock Vina. **(E**–**G)** Left: distribution of high binding energy hits by LeDock; right: overview of the interactions between the ligand and the receptor structure.

During the first round of virtual screening, we abandoned the process for nsp3 in advance due to the impracticable binding energy of compounds. Other targets had a lot of ligands whose binding energies were lower than −9 kcal/mol (Figures 1A–D). After the second round of virtual screening, we observed that NTPase possesses the most hits among all targets (Figures 1E–G). It indicated that PEDV NTPase was a potential target by the small compounds, so we focused on the compounds targeting PEDV NTPase for further research (Table 3).

**TABLE 3:** Summary of compounds with high binding energy to PEDV NTPase.

Name	LeDock	Vina	Lipinski	Pfizer	In sale
ZINC000226650999	−11	−9.8	—	●	●
ZINC000002192336	−10.5	−9.7	—	●	●
ZINC000150342276	−10.3	−9.5	—	●	—
ZINC000245241447	−10.3	−9.5	—	●	●
ZINC000245241873	−10.3	−9.7	—	●	●
ZINC000079232074	−10.1	−9.8	—	●	●
ZINC000085593135	−10.1	−9.9	—	●	—
ZINC000824692125	−10.1	−9.7	—	●	●
ZINC000004147756	−9.99	−9.8	●	●	●
ZINC000002161529	−9.92	−10.2	●	●	●
ZINC000012899676	−9.9	−9.5	●	●	●
ZINC000004147757	−9.87	−9.6	—	●	●
ZINC000004259786	−9.84	−10.4	●	●	●
ZINC000004201042	−9.82	−9.5	—	●	—
ZINC000002313433	−9.81	−9.6	●	—	●
ZINC000004259858	−9.77	−9.6	—	●	—
ZINC000002131967	−9.74	−9.5	●	●	●
ZINC000004147789	−9.73	−9.9	—	●	—
ZINC000000637802	−9.69	−9.5	●	●	●
ZINC000102986418	−9.66	−9.9	—	●	—
ZINC000524729130	−9.66	−9.9	—	●	●
ZINC000263586742	−9.63	−9.6	—	●	●
ZINC000003666978	−9.56	−9.5	—	●	●
ZINC000004259775	−9.55	−10.9	—	●	●
ZINC000004259769	−9.53	−10.2	●	●	●
ZINC000085593156	−9.51	−9.8	—	●	—

### ZINC12899676 Inhibits the Activity of PEDV NTPase

**FIGURE 2:**
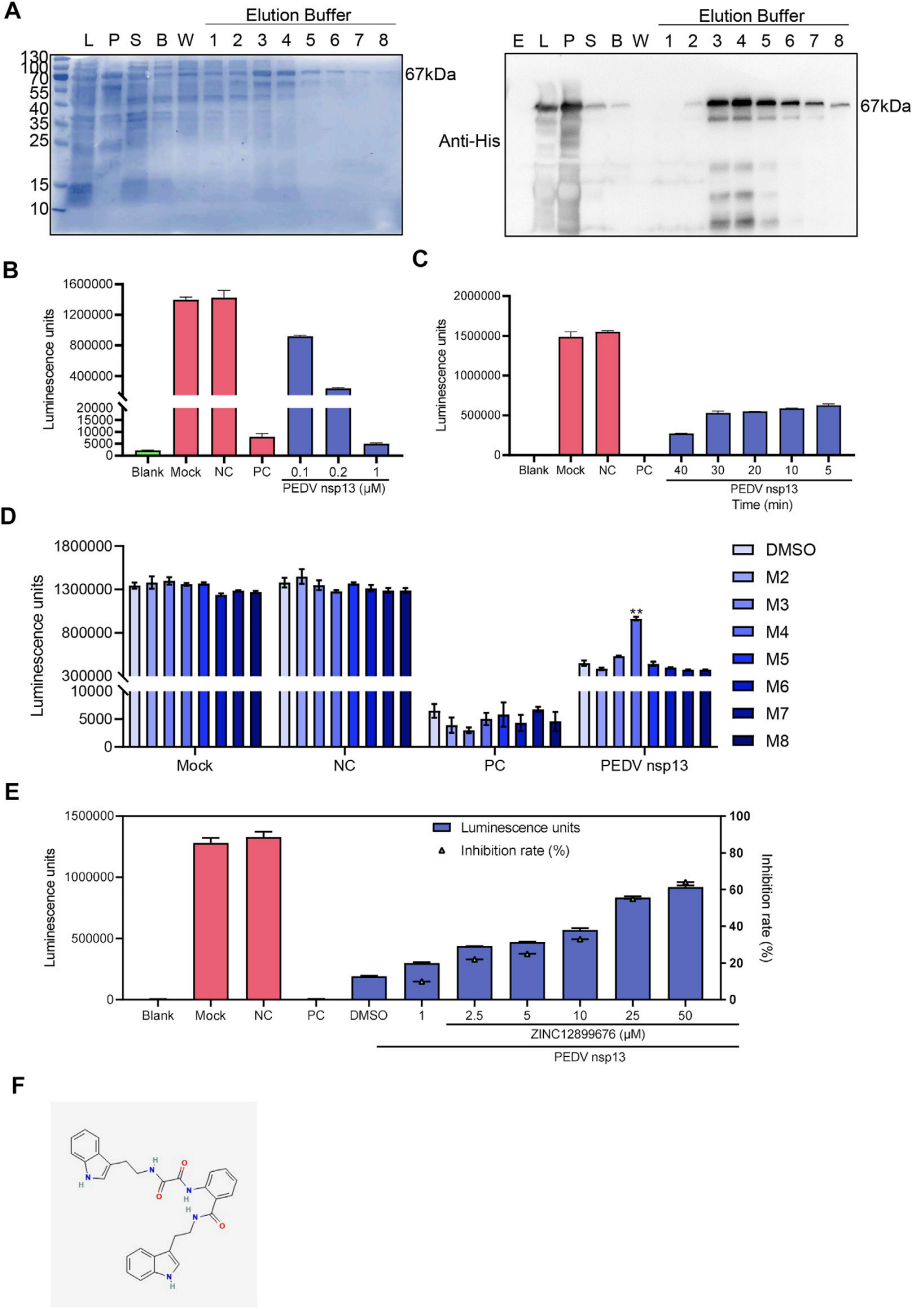
ZINC12899676 inhibits the activity of PEDV NTPase. **(A)** Purification of PEDV nsp13. “E” represents empty vector, “L” represents whole bacteria after induction, “S” represents the supernatant after ultrasonication, “B” represents the liquid collected after washing using binding buffer, “W” represents the liquid collected after washing using washing buffer, and numbers (1–8) represent the liquid collected after washing using elution buffer. **(B)** Purified nsp13 recombinant proteins (0, 0.1, 0.2, or 0.5 μM) were incubated with ATP in the reaction buffer at 37°C for 20 min, and then, the Kinase-Glo reagent mix was added, and the ATPase activity was measured. **(C)** PEDV nsp13 (0.2 μM) was reacted with ATP at 37°C for 5, 10, 20, 30, or 40 min, and then, the ATPase activity was measured. **(D)** Compounds (25 μM) or DMSO were added to the reaction mixture 10 min ahead of ATP, and the ATPase activity was measured. **(E)** Different concentrations of ZINC12899676 were added to the reaction mixture 10 min ahead of ATP, and the ATPase activity was measured. **(F)** 2D structure of ZINC12899676.

Considering binding energy and drug-like characteristic of compounds, seven small compounds, namely, M2 to M8, were selected to ascertain the effect of compounds to PEDV NTPase (Table 4). We first conducted the prokaryotic expression and purification of PEDV nsp13 (Figure 2A) and detected the ATPase activity of recombinant nsp13 proteins by using a Kinase-Glo Plus Luminescent Kinase Assay kit, which measured luminescence caused by the remaining ATP in the reaction mixture. The reaction consisting PEDV N protein as a negative control remained a high luminescent unit same as the mock group. Apyrase from potatoes as a positive control decreased the luminescent unit to little. The amount of ATP remaining in the reaction mixture sharply decreased as the PEDV nsp13 concentration increased (0.1, 0.2, and 1 μM) (Figure 2B). When nsp13 was added, the ATP immediately decreased along with time (Figure 2C). It indicated that recombinant nsp13 possessed the ATP hydrolysis activity.

**TABLE 4:** IUPAC name and 2D structure of selected compounds.

No	Name	IUPAC name	2D structure
M2	ZINC79232074	7-[(2R)-3-[furan-2-ylmethyl-[(2R)-2-hydroxy-3-(8-methyl-2-oxo-4-phenylchromen-7-yl)oxypropyl]amino]-2-hydroxypropoxy]-8-methyl-4-phenylchromen-2-one	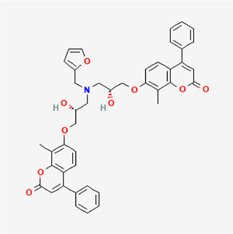
M3	ZINC2161529	(2R)-2-[[2-(3-benzyl-6-chloro-4-methyl-2-oxochromen-7-yl)oxyacetyl]amino]-3-(5-hydroxy-1H-indol-3-yl)propanoic acid	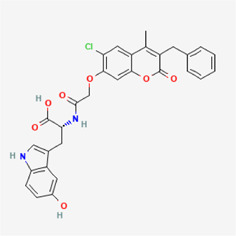
M4	ZINC12899676	N-[2-(1H-indol-3-yl)ethyl]-N'-[2-[2-(1H-indol-3-yl)ethylcarbamoyl]phenyl]oxamide	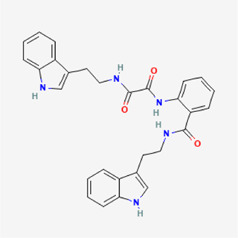
M5	ZINC2313433	4-[(4-chlorophenyl)sulfanylmethyl]-2-(2-oxo-2-phenothiazin-10-ylethyl)sulfanyl-1H-pyrimidin-6-one	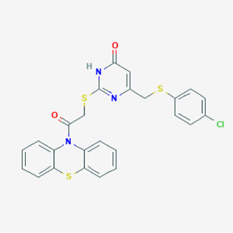
M6	ZINC2131967	(2R)-2-[[2-(4-ethyl-8-methyl-2-oxochromen-7-yl)oxyacetyl]amino]-3-(5-hydroxy-1H-indol-3-yl)propanoic acid	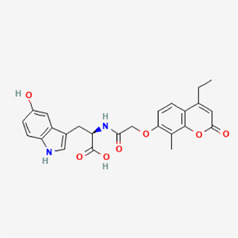
M7	ZINC524729130	(3R)-3-(4-hydroxy-3-methoxyphenyl)-3-(3-hydroxy-6-methyl-4-oxopyran-2-yl)-N-[2-(10-oxo-2,3,7,11-tetrazatricyclo[7.4.0.02,6]trideca-1(9),3,5,7,12-pentaen-11-yl)ethyl]propanamide	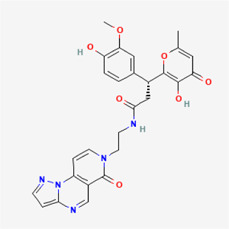
M8	ZINC263586742	2-[[(7S)-7-acetamido-1,2,3-trimethoxy-9-oxo-6,7-dihydro-5H-benzo[a]heptalen-10-yl]amino]-N-[2-(2-methyltetrazol-5-yl)phenyl]acetamide	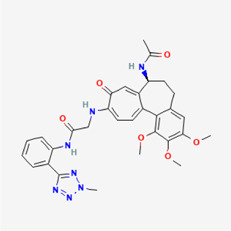

Compounds (25 μM) or DMSO were added to the reaction mixture 10 min ahead of ATP to measure the inhibition of compounds to the activity of PEDV NTPase, and then, we found that M4 (ZINC12899676) increased the remaining ATP compared with DMSO. In other words, ZINC12899686 significantly inhibited the activity of PEDV NTPase (Figure 2D). PEDV nsp13 was treated with different concentrations of ZINC12899676 and, then the inhibition rate was calculated. The percentage of inhibition was calculated as follows: percentage of inhibition (%) = 100 × [1 − RLU of (negative control–drug treatment)/RLU of (negative control–DMSO treatment)]. ZINC12899676 inhibited the activity of PEDV NTPase in a dose-dependent manner (Figures 2E,F). The IUPAC of ZINC12899676 is N-[2-(1H-indol-3-yl)ethyl]-N'-[2-[2-(1H-indol-3-yl)ethylcarbamoyl]phenyl]oxamide.

### Active Pocket of PEDV NTPase Is Targeted by ZINC12899676 Strongly and Its Conformational Change Occurs

**FIGURE 3:**
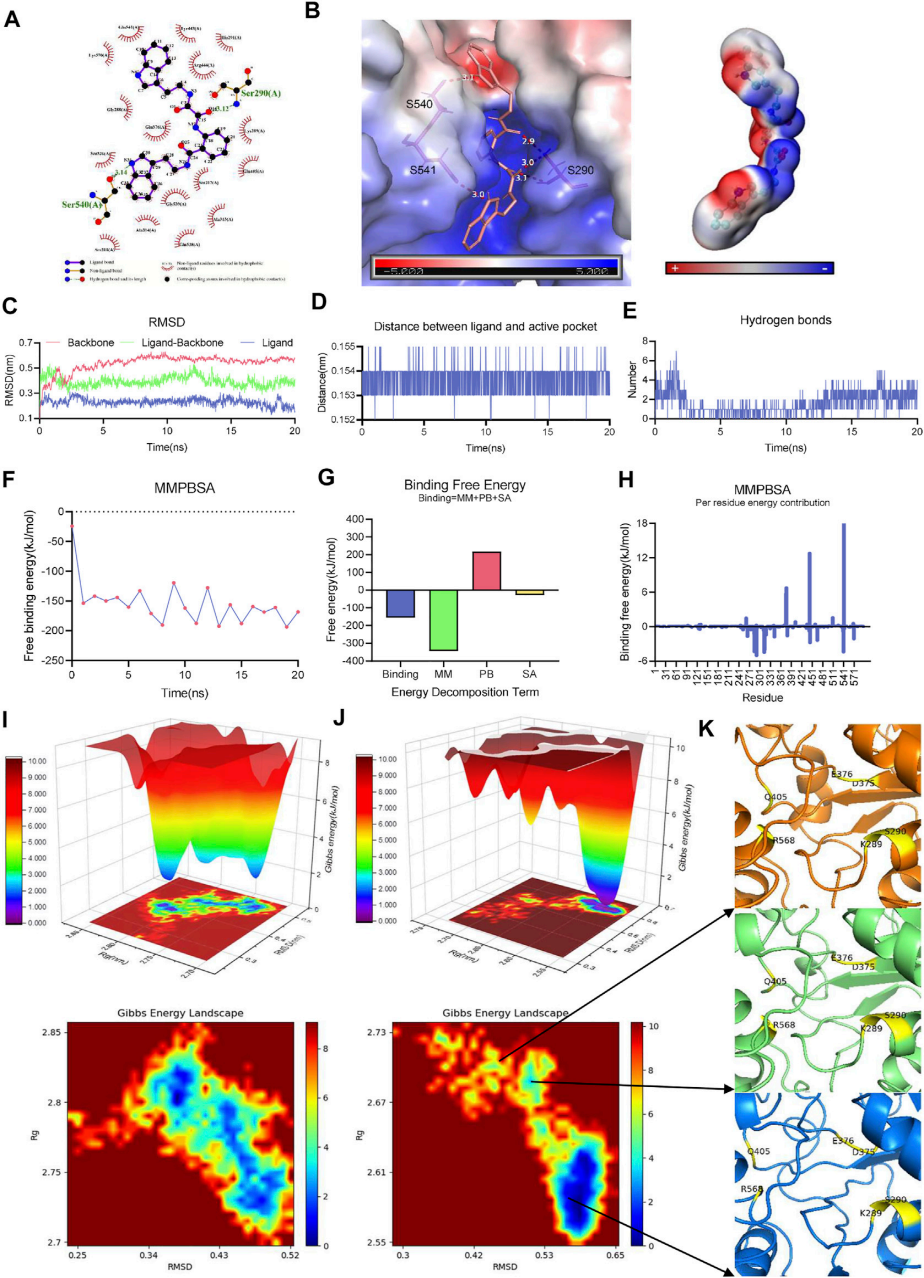
Active pocket of PEDV NTPase is targeted by ZINC12899676 strongly, and its conformational change occurs. **(A)** Interactions between PEDV NTPase and ZINC12899676. Intermolecular hydrogen bonds are shown by green dashed lines with distance in Å. Non-ligand residues in hydrophobic contacts are shown by red gear. **(B)** Left: H-bond between PEDV NTPase and ZINC12899676 analyzed by Pymol and the electrostatic potential surface representation of PEDV NTPase in complex with ZINC12899676; right: electrostatic potential surface of ZINC12899676. **(C)** RMSD values of protein backbone, ligand-backbone, and ligand over the simulation time. **(D)** Distance between the ligand and active pocket of PEDV NTPase over the simulation time. **(E)** Number of H-bonds involved in the interaction between the protein and compound during the MD simulation. **(F)** Free binding energy of PEDV NTPase and ZINC12899676 calculated by MM-PBSA. **(G)** Energy decomposition of the binding energy calculated by MM-PBSA. **(H)** Decomposition of the energy per residue in the interaction between PEDV NTPase and ZINC12899676 calculated by MM-PBSA. The Gibbs energy landscape obtained during 20 ns MD simulation for **(I)** free PEDV NTPase and **(J)** PEDV NTPase-ZINC12899676 complex. **(K)** Conformation of the active pocket during different periods.

To figure out the mechanism of ZINC12899676 against PEDV NTPase, we analyzed the molecular docking between PEDV NTPase and its inhibitor. Hydrogen bonds are generally considered to be important contributors to protein–ligand binding (Chen et al., 2016). LigPlot software visualized the two H-bonds and some residues involving the hydrophobic interaction between the ligand and the receptor (Figure 3A). The H-bond between the compound and the key active site S290 was also visualized by Pymol. These hydrogens bonds and hydrophobic force may contribute to the binding of the ligand and receptor. The various numbers of hydrogen bonds between LigPlot and Pymol may be due to the different judgment standards. In drug discovery, it is widely accepted that electrostatic potential (ESP) surface complementarity between the protein and ligand is critically important in order to obtain optimal affinity and selectivity (Rathi et al., 2020). The ESP surfaces of PEDV NTPase and ZINC12899676 were respectively calculated by APBS plugin integrated into Pymol and Gaussian. The protein ESP surfaces used blue to represent the positive electrostatic potential and red to represent the negative electrostatic potential, and the color representation ligand electrostatic potential surface was the opposite. We could observe that the head and backside of ligand ESP surfaces were fully compatible with the protein ESP surfaces in relevant position, which explained the ZINC12899676 and PEDV NTPase interaction using quantum mechanics (Figure 3B).

MD utilizes Newtonian physics to simulate atomic movements in a solvated system and is an accurate computational method for simulating protein–drug interactions (Hollingsworth and Dror, 2018). To assess receptor and ligand stability, a 20 ns molecular dynamic simulation was carried out. The smooth and steady RMSD of the backbone/ligand indicated less structural changes during MD simulation. The protein–compound complex reached its stability throughout the simulation with relative RMSD in acceptable range, with few fluctuations observed (Figure 3C). Distances between compound and the active pocket of NTPase were highly conserved and tight during simulation calculations (Figure 3D). We monitored the number of hydrogen bonds during the MD simulations to better capture the intermolecular polar interactions. The stable existence of H-bonds in the complex began with the 12 ns, which explained the integrity and stable nature of the protein–ligand complex (Figure 3E). In order to ascertain the observed stability, the binding affinities of ZINC12899676 with the NTPase complex were defined by binding free energy based on the molecular mechanics Poisson–Boltzmann surface area (MM-PBSA) approach and measured using the gmx_mmpbsa tool. The estimated MM-PBSA binding energy free energy variation with time is shown in Figure 3F, which suggested the efficient and steady binding of the ligand to NTPase. The average of binding free energy of the protein–ligand complex obtained using MM-PBSA was −154.702 kJ/mol and indicated a very strong affinity (Figure 3G). Residues which provided free binding energy were mainly located in the active pocket of PEDV NTPase, which was consistent with the actual interaction situation (Figure 3H).

The Gibbs free energy landscape (FEL) is analyzed for understanding the conformational transition beneath the protein–compound interaction, using the reaction coordinates of RMSD and Rg, respectively (Efaz et al., 2021). FELs of PEDV NTPase and the complex system of NTPase in presence of ZINC12899676 were displayed in 2D and 3D graphs. The single energy cluster with deep blue plots in the energy distribution indicated strong and stable interaction, and multiple energy minimum clusters with shallow yellow plots reflected weak and unstable interaction of the protein and ligand. The deep blue areas with minimal energy were found to be highly concentrated in NTPase in the presence of the ZINC12899676 complex (Figure 3J) when compared to the NTPase alone (Figure 3I). This result elucidated that the compound had the potential to induce NTPase to enter the local energy minimal state and to form the stable binding of the compound at the protein active pocket. Furthermore, the different stage conformations during the energy minimal state had been extracted from the trajectory. It was obvious that the β-sheet located in R568 had been transformed into α-helix, and Q405 shifting out from the active pocket led to the expansion of pocket (Figure 3K). The conformation change caused by ZINC12899676 may finally lead to the dysfunction of NTPase. In general, the ligand strongly binding to enzyme’s active site and transforming the active pocket conformation contributed to inhibit the activity of PEDV NTPase.

### ZINC12899676 Has Antiviral Activity Against PEDV *In Vitro*

**FIGURE 4:**
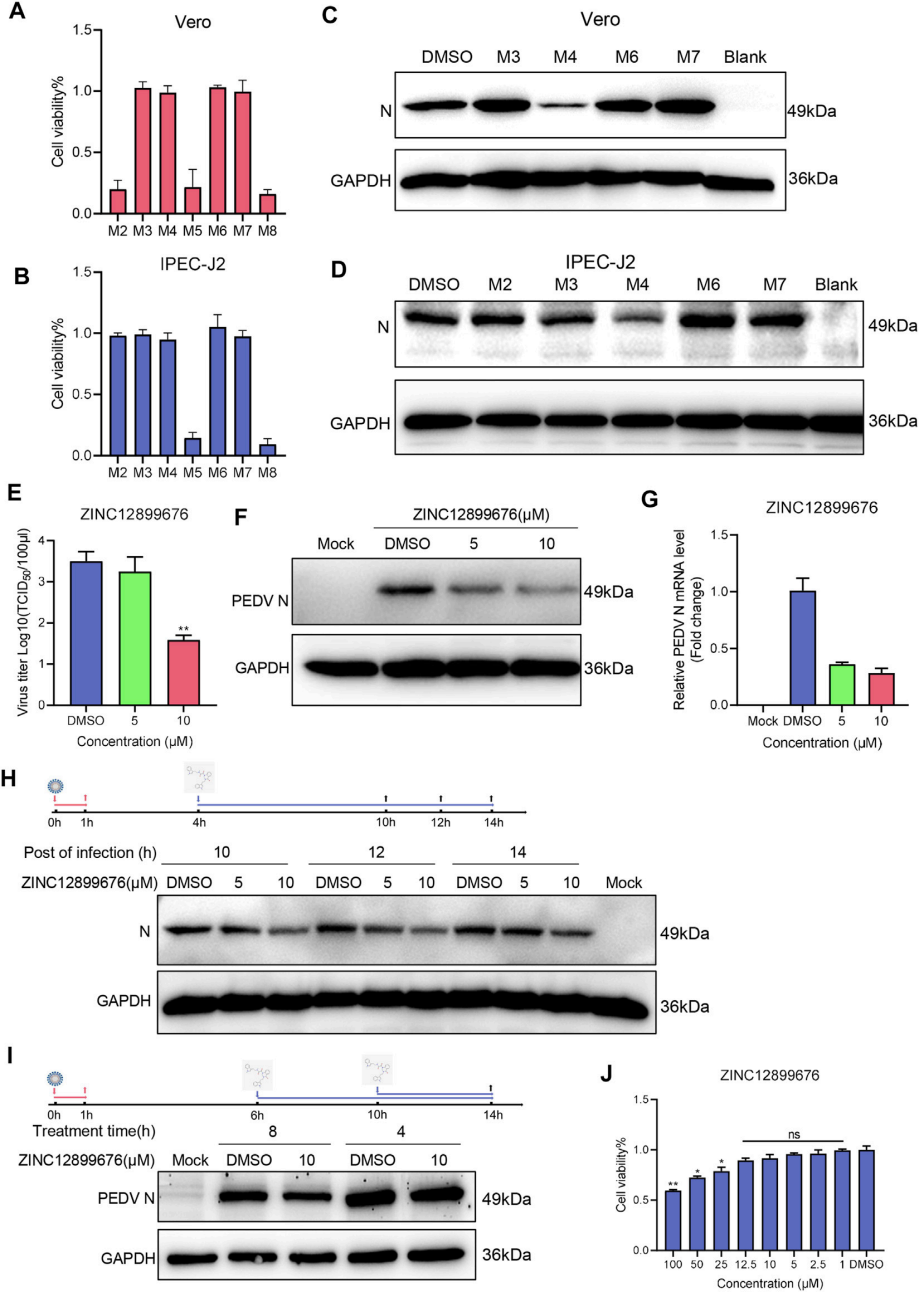
ZINC12899676 has antiviral activity against PEDV *in vitro*. **(A**,**B)** Cell viability of Vero and IPEC-J2 cells pretreated with compounds (10 μM) and incubated for 24 h. The results are from one of three independent experiments. **(C**,**D)** Vero and IPEC-J2 cells were pretreated with compounds (10 μM) for 1 h and then infected with PEDV for 1 h at 37°C. The cells were washed with PBS and then incubated in a fresh medium containing compounds for 16 h. DMSO served as the treatment control. **(E**–**G)** IPEC-J2 cells were pretreated with the indicated concentrations of ZINC12899676 for 1 h and then infected with PEDV for 1 h at 37°C. The cells were washed with PBS and then incubated in fresh medium containing ZINC12899676 for 16 h. DMSO served as the treatment control. **(E)** Culture supernatants were collected at indicated time points for viral titration. Results are expressed as TCID_50_. Titers from three independent experiments are shown as means ± SD (error bars). **(F)** Western blot for the viral N-protein in cells infected with PEDV and treated with indicated concentrations of ZINC12899676 or DMSO, at 16 hpi. **(G)** Relative PEDV N mRNA levels, determined by qRT-PCR, and expressed relative to that in DMSO-treated cells. The internal loading control was GAPDH. **(H)** IPEC-J2 cells were incubated with PEDV at 37°C for 1 h and washed three times with PBS to remove free virus. At 4 hpi, the culture medium was replaced with fresh DMEM containing ZINC12899676 or DMSO, and the cultures were incubated at 37°C. PEDV N and GAPDH proteins in the samples collected at 10, 12, and 14 hpi were measured using Western blot. **(I)** IPEC-J2 cells were incubated with PEDV at 37°C for 1 h and washed three times with PBS to remove free virus. At 6 or 10 hpi, the culture medium was replaced with fresh DMEM containing ZINC12899676 or DMSO, and the cultures were incubated at 37°C. PEDV N and GAPDH proteins in the samples collected at 14 hpi were measured using Western blot. **(J)** Cell viability of IPEC-J2 cells pretreated with indicated concentrations of ZINC12899676 and incubated for 24 h.

Since the compound inhibited the activity of PEDV NTPase, we further evaluated its anti-PEDV activity *in vitro*. Some compounds were ruled out because of the visible cytotoxicity (Figures 4A,B), and the remaining compounds were screened by Western blot. Results demonstrated that only ZINC12899676 had anti-PEDV activity coinciding with the inhibition of the NTPase enzymatic activity (Figures 4C,D).

To elucidate the antiviral activity of ZINC12899676 in porcine cells, IPEC-J2 cells were treated with 5 and 10 μM compounds for 1 h and then infected with PEDV (1 MOI). TCID_50_, Western blot, and qRT-PCR analysis showed that the virus titers, N-protein, and mRNA levels significantly decreased in a dose-dependent manner (Figures 4E–G). We examined the effect of tomatidine on PEDV replication by adding the compound during the replication stage. As shown in Figure 4H, ZINC12899676 treatment significantly decreased PEDV N protein levels compared to treatment with DMSO, suggesting that the compound inhibits PEDV replication. In addition, ZINC12899676 was added in different hours post-infection. As the action time of ZINC12899676 on replication stage was reduced, the antiviral effect of the drug decreased (Figure 4I). Inhibition of the activity of the PEDV NTPase responsible for replication corresponded to the action on the replication stage of PEDV by ZINC12899676. Meanwhile, the concentrations of the compound exerting on IPEC-J2 cells displayed no cytotoxicity through cell viable assay (Figure 4J). In summary, ZINC12899676 had been proved to fight against PEDV *in vitro*.

### Neural Network Is Built to Accelerate the Discovery of Potential Compounds Targeting PEDV NTPase

**FIGURE 5:**
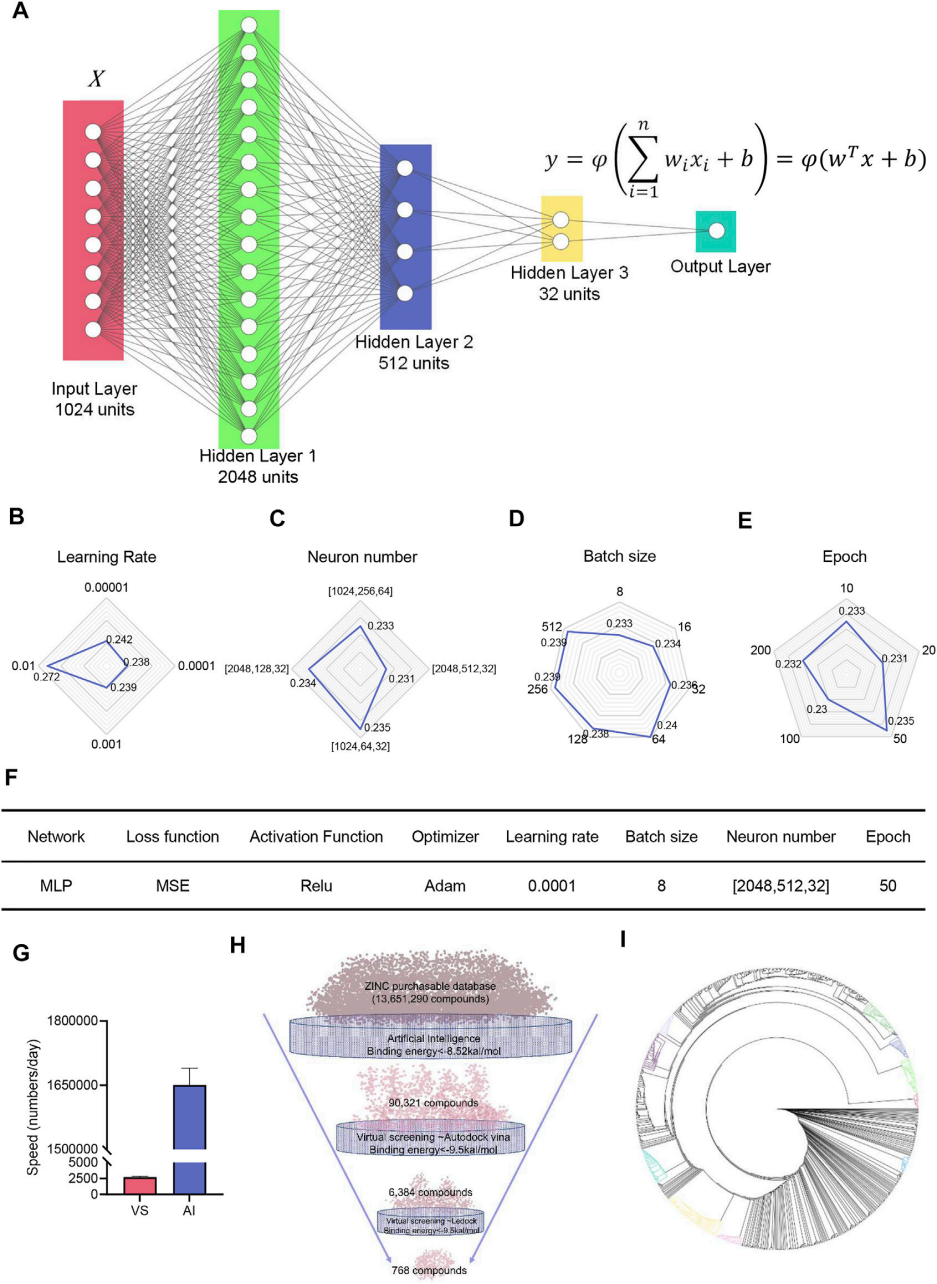
Neural network built to accelerate the discovery of potential compounds targeting PEDV NTPase. **(A)** Structure of the deep neural network. Neurons are represented by circles. **(B**–**E)** Tunning of hyperparameters. **(F)** Parameter and hyperparameter settings for the deep neural network. **(G)** Comparison of the speed of virtual screening with artificial intelligence. **(H)** Combination application of AI and VS to find compounds with high binding energy to PEDV NTPase. **(I)** Clusters of compounds targeting PEDV NTPase with high binding energy.

To improve the structure diversity of lead compounds, we had meant to conduct an ultra-large virtual screening to discover more lead compounds. Structure-based virtual screening costs too many computes. Recently, deep learning (DL) models show promising performance for drug property prediction. We built a simple drug property prediction multilayer perceptron (MLP) neural network to exclude the low binding energy compounds, thereby narrowing the scope of virtual screening (Figure 5A). The remaining high binding energy compounds through AI prediction were carried out by two-round virtual screening. After the tunning of hyperparameters, the MSE finally reached to 0.23 (Figures 5B–E). The parameters and hyperparameters are shown in Figure 5F. The speed of neuron network prediction was more than 600 times than virtual screening (Figure 5G); it indicated neuron network predicting had the capacity to screen millions of compounds. We finally quickly discovered 768 compounds with high binding energy (<−9.5 kcal/mol) from 13,651,290 compounds through a workflow: AI predicting-AutoDock Vina-LeDock virtual screening (Figure 5H). Cluster analysis based on the structures was performed on these compounds by ChemMine software (Backman et al., 2011). We can observe that the molecules were grouped into several clusters (Figure 5I). The top item from each cluster should be selected as the representative of the related cluster and used for more perspective of PEDV NTPase inhibitors. This result may be beneficial to enhance the structure diversity of lead compounds targeting PEDV NTPase.

## Discussion

Researchers have found several antiviral small compounds against PEDV *in vitro*. There are some compounds such as salinomycin (Yuan et al., 2021), homoharringtonine (Dong et al., 2018), and A77 1726 (Li X. et al., 2020) preventing PEDV proliferation by affecting the host defense system. Many compounds inhibit PEDV replication by targeting PEDV 3CLpro, such as tomatidine (Wang et al., 2020), hypericin (Zhang Y et al., 2021), quercetin (Li Z. et al., 2020), and GC376 (Ye et al., 2020). PEDV RdRp also can be the target of antiviral drugs (Xie et al., 2021). In this study, a natural compound ZINC12899676 was first identified to significantly inhibit the NTPase activity, and it significantly inhibited PEDV replication *in vitro*.

The nsp13s from representative α- and β-coronavirus shared conserved helicase and NTPase. It possesses NTPase and helicase activities (Jia et al., 2019; Ren et al., 2021). Coronavirus NTPase can hydrolyze nucleoside triphosphate in the presence of Mg^2+^ and Mn^2+^ to produce energy, which can be utilized by helicase for unwinding dsDNA and dsRNA (Ivanov et al., 2004). Thus, we speculated ZINC12899676 can also inhibit PEDV nsp13 helicase activity by limiting energy production (Yang et al., 2007). Coronavirus nsp13 can make an impact on host defense. Researchers reported that the host interferon signaling pathway can be suppressed by SARS-CoV-2 nsp13 (Lei et al., 2020; Yuen et al., 2020; Feng et al., 2021). In addition, SARS-CoV-2 nsp13 hijacks host deubiquitinase UPS13 and confronts host antiviral immune response (Guo et al., 2021). Inflammasome complexes have both positive and negative effects on the host. SARS-CoV-2 nsp13 domains responsible for helicase activity significantly reduced NLRP3-inflammasome-induced caspase-1 activity and IL-1β secretion (Kim et al., 2021). Many DNA and RNA viruses can cause disturbance of cell cycle regulation; researchers demonstrated that the interaction between nsp13 and DNA polymerase δ induced DNA replication stress in IBV-infected cells (Xu et al., 2011). Therefore, it is extremely meaningful to seek small compounds targeting PEDV NTPase.

So far, a variety of docking programs are available for the scientific community. These programs have been systematically evaluated by analyzing the accuracies of binding pose prediction and binding affinity estimation, which are two pillars for a successful docking experiment (Saikia and Bordoloi, 2019). Results showed that LeDock had the best sampling power among the five academic programs, and AutoDock Vina had the best scoring power among the commercial and academic programs (Wang Z et al., 2016). Therefore, we carried out virtual screening by AutoDock Vina and LeDock programs. According to the report, it is inevitable to generate false-negatives and positives during virtual screening (Ferreira et al., 2010). Furthermore, expectation of identifying high-affinity compounds by VS and subjectivity of post-VS compound selection are two pitfalls in virtual screening. Avoiding these two problems may better optimize the efficiency and accuracy of virtual screening (Scior et al., 2012). Structure-based virtual screening costs too many computes. The speed of virtual screening is not sufficient to face the billion drugs in the database. Artificial intelligence (AI) has recently demonstrated its superior performance than classic methods to assist computational drug discovery, owing to its expressive power in extracting, processing, and extrapolating patterns in molecular data (Ekins et al., 2019; Zhavoronkov et al., 2019; Stokes et al., 2020).

The 3D structure of targets is the fundamental of virtual screening. We determined protein structures through comparing various modeling approaches and assessing the reliability of modeling structures. We predicted protein structures by I-TASSER, SWISS-MODEL, and trRosetta, which have been widely used and granted numerous awards. Benefits from the determination of the crystal structure of SARS-CoV-2 (Malone et al., 2021; Newman et al., 2021), SWISS-MODEL, and trRosetta-based homology modeling have better performance. During our preparation of this article, Alphafold2 (Jumper et al., 2021), and RoseTTAFold (Baek et al., 2021), delivering a revolutionary advance for protein structure predictions, have been published (Mullard, 2021). The combination of these tools can create 3D models of the eukaryotic protein complex which may help accelerate the development of future novel therapeutics and drug discoveries (Humphreys et al., 2021). However, there is lack of online AlphaFold service to utilize, and it demands ultra-high hardware to deploy locally. Reassuringly, we selected the PEDV NTPase structure modeled by trRosetta, which showed performance similar to AlphaFold (Pakhrin et al., 2021). In addition, other researchers revealed the enzyme catalytic center of several coronavirus replicative enzymes. The enzyme catalytic centers of nsp3 are C1729, H1888, D1901, and W1730 (Durie et al., 2021). Residues H41 and C144 are key amino acids of the activity of nsp5 (Wang D et al., 2016). Amino acid residues G608, W609, D610, Y611, L750, S751, D752, D753, K790, C791, W792, E803, F804, C805, and S806 form the active pocket of nsp12 (Aftab et al., 2020). The active pocket of nsp13 NTPase is constituted by K289, S290, D375, E376, Q405, and R568 (Mirza and Froeyen, 2020). The active pocket of nsp15 is composed of H226, H241, and K282 (Wu et al., 2020). These findings contributed to the selection of the PEDV enzyme active pocket.

The scale of natural products with numerous structural and chemical diversities is much larger than any synthetic libraries of small molecules (Shen, 2015). The utilization of natural products and/or synthetic variations using their novel structures from 1981 to 2019 is still alive and well (Newman and Cragg, 2020). At the same time, researchers found PEDV replication can be inhibited by natural compounds, such as griffithsin (Li et al., 2019), coumarin (Yang J. L. et al., 2015), and so on. Thus, natural products library of ZINC15 was selected for virtual screening. Delightfully, ZINC12899676 showed drug-like properties in accordance with Lipinski’s rule of five and Pfizer’s rule. In addition, ZINC12899676 was previously reported to inhibit TLR1-TLR2 heterodimerization as an immunomodulatory agent (Zhong et al., 2015).

In conclusion, we observed that PEDV NTPase had the most hits among the five targets (PLP2, 3CLpro, RdRp, NTPase, and NendoU) through two-round virtual screening. ZINC12899676 was identified to significantly inhibit the NTPase activity. Computational biology analysis indicated that the active pocket of PEDV NTPase was targeted by ZINC12899676 strongly, and its conformational change occurred. Furthermore, ZINC12899676 significantly inhibited PEDV replication *in vitro*. Our findings first demonstrated that ZINC12899676 inhibits PEDV replication by targeting NTPase, and then, NTPase may serve as a novel target for anti-PEDV.

## Data Availability

The original contributions presented in the study are included in the article/Supplementary Material, further inquiries can be directed to the corresponding author.
