# Supplementary figures and images for: Natural Compound ZINC12899676 Reduces Porcine Epidemic Diarrhea Virus Replication by Inhibiting the Viral NTPase Activity
Source: Front Pharmacol. 2022 May 4;13:879733. doi: 10.3389/fphar.2022.879733 (PMC9114645; doi:10.3389/fphar.2022.879733)

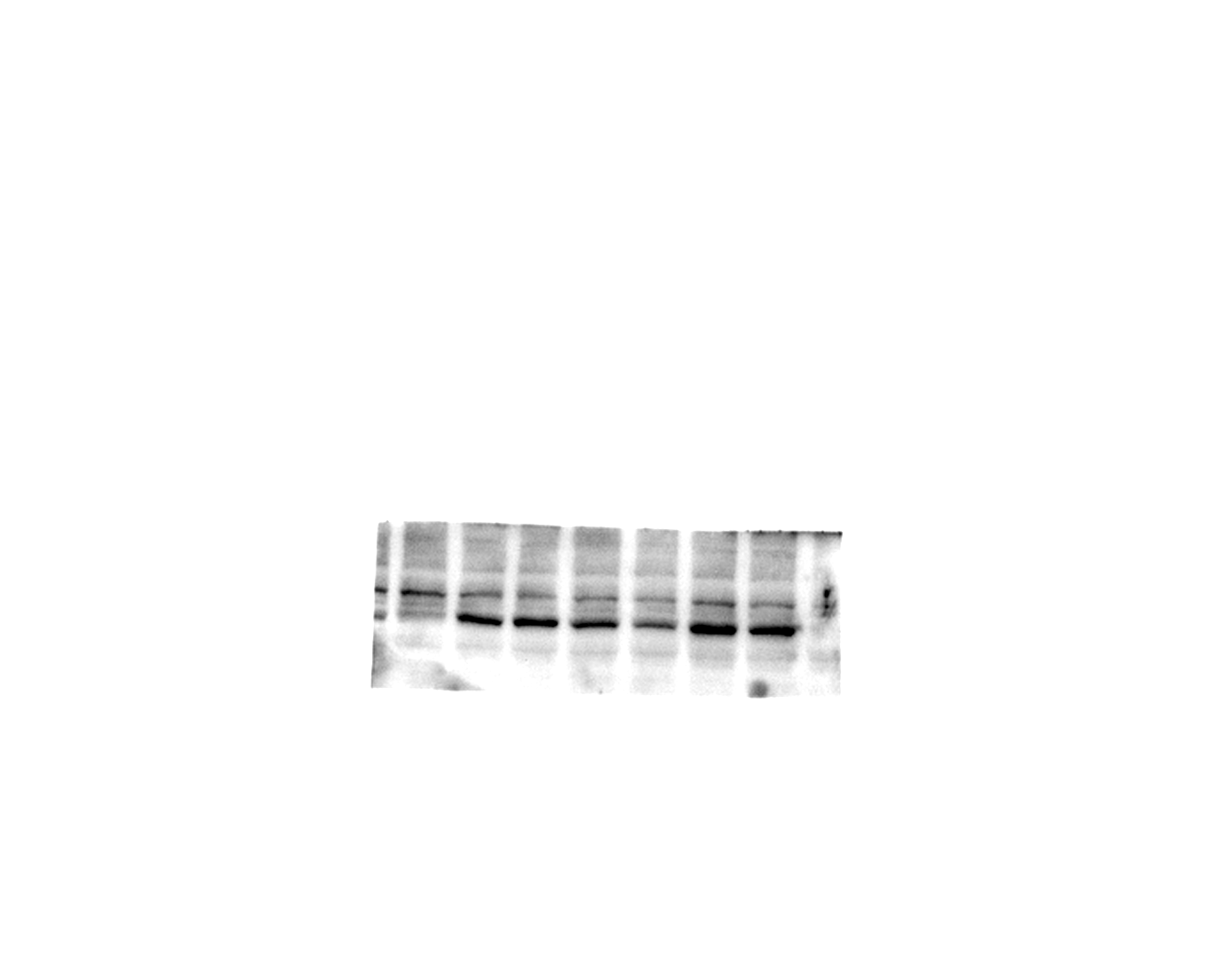

Supplement: Supplementary file 1 [file Image6.TIF]

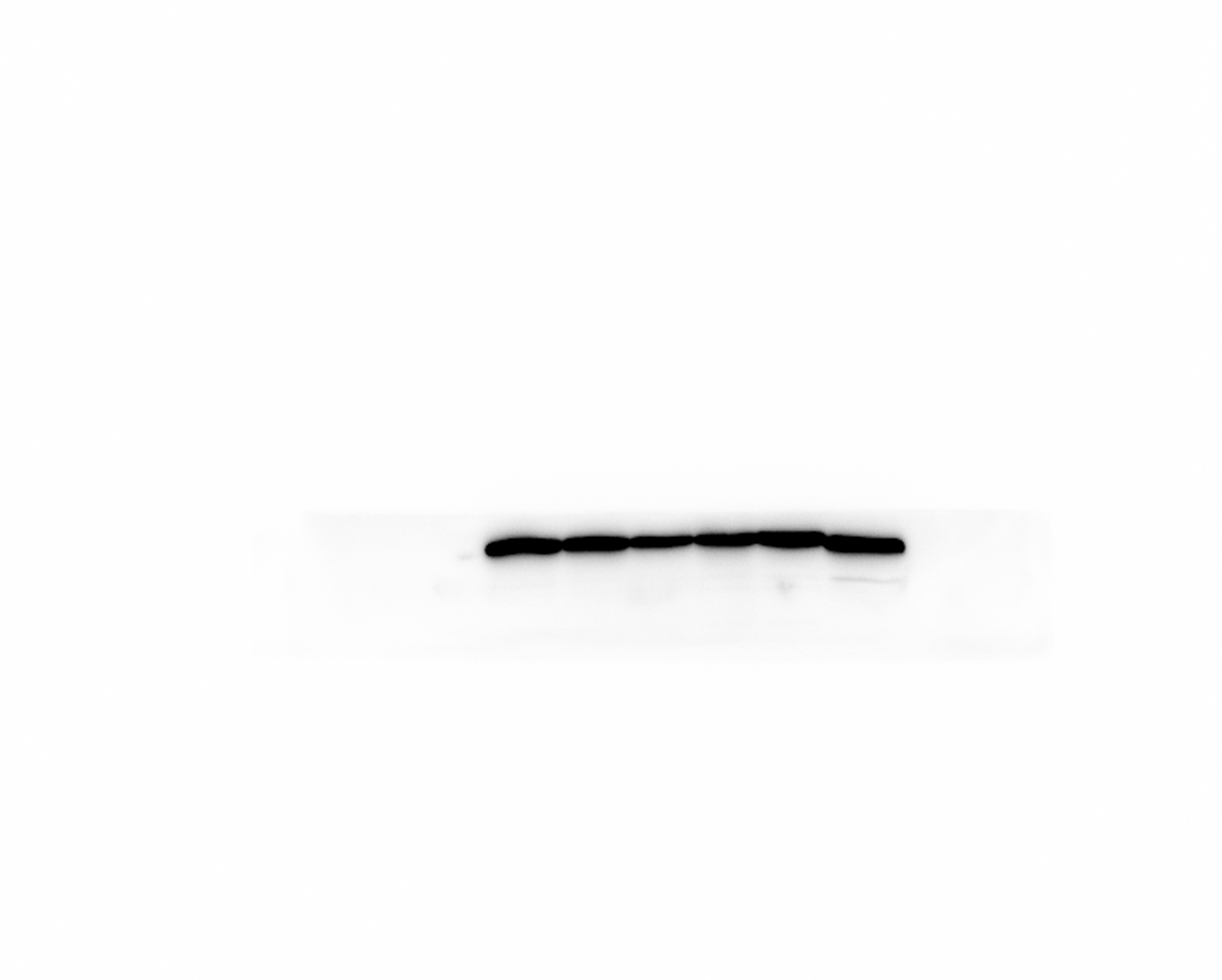

Supplement: Supplementary file 2 [file Image3.TIF]

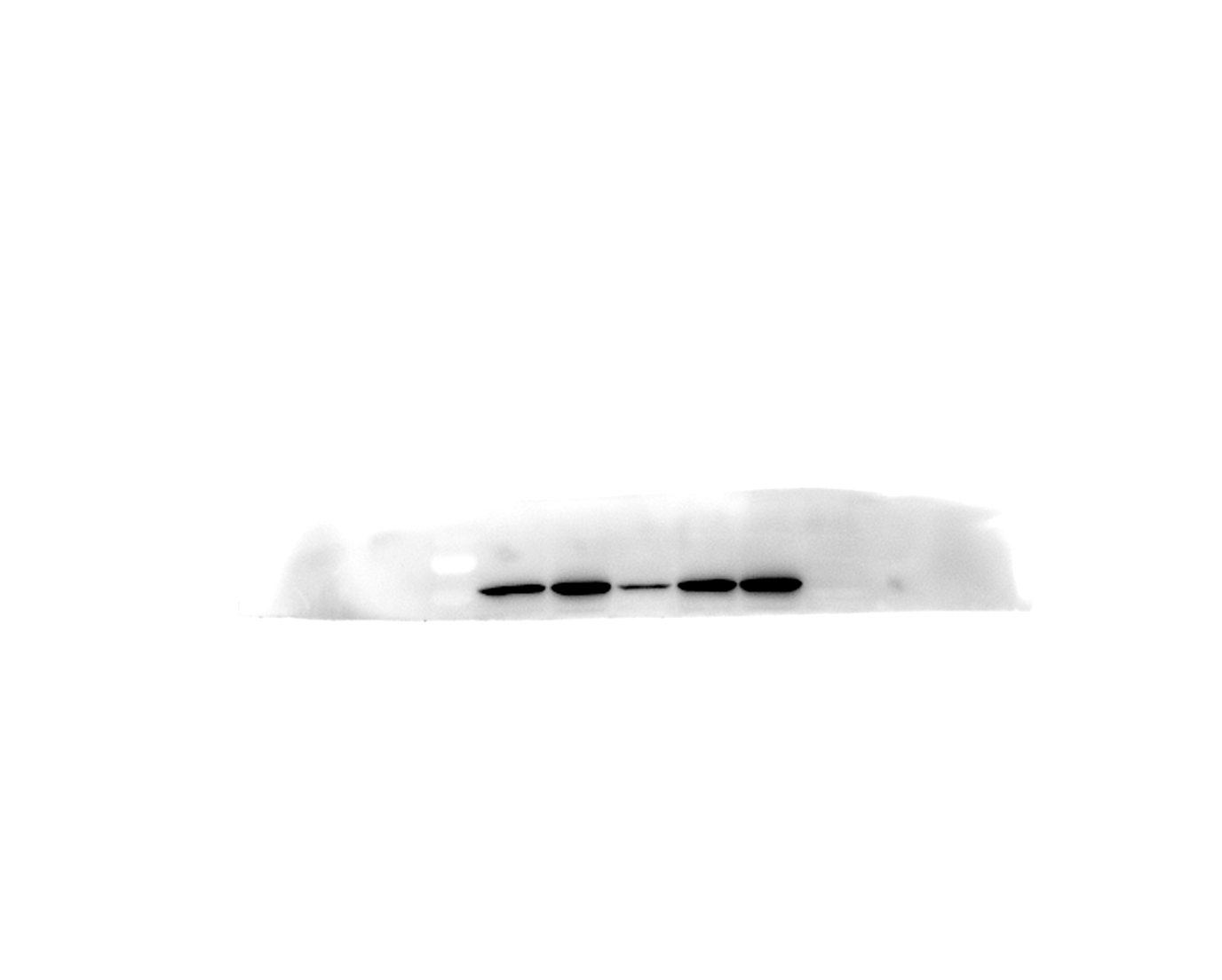

Supplement: Supplementary file 3 [file Image4.TIF]

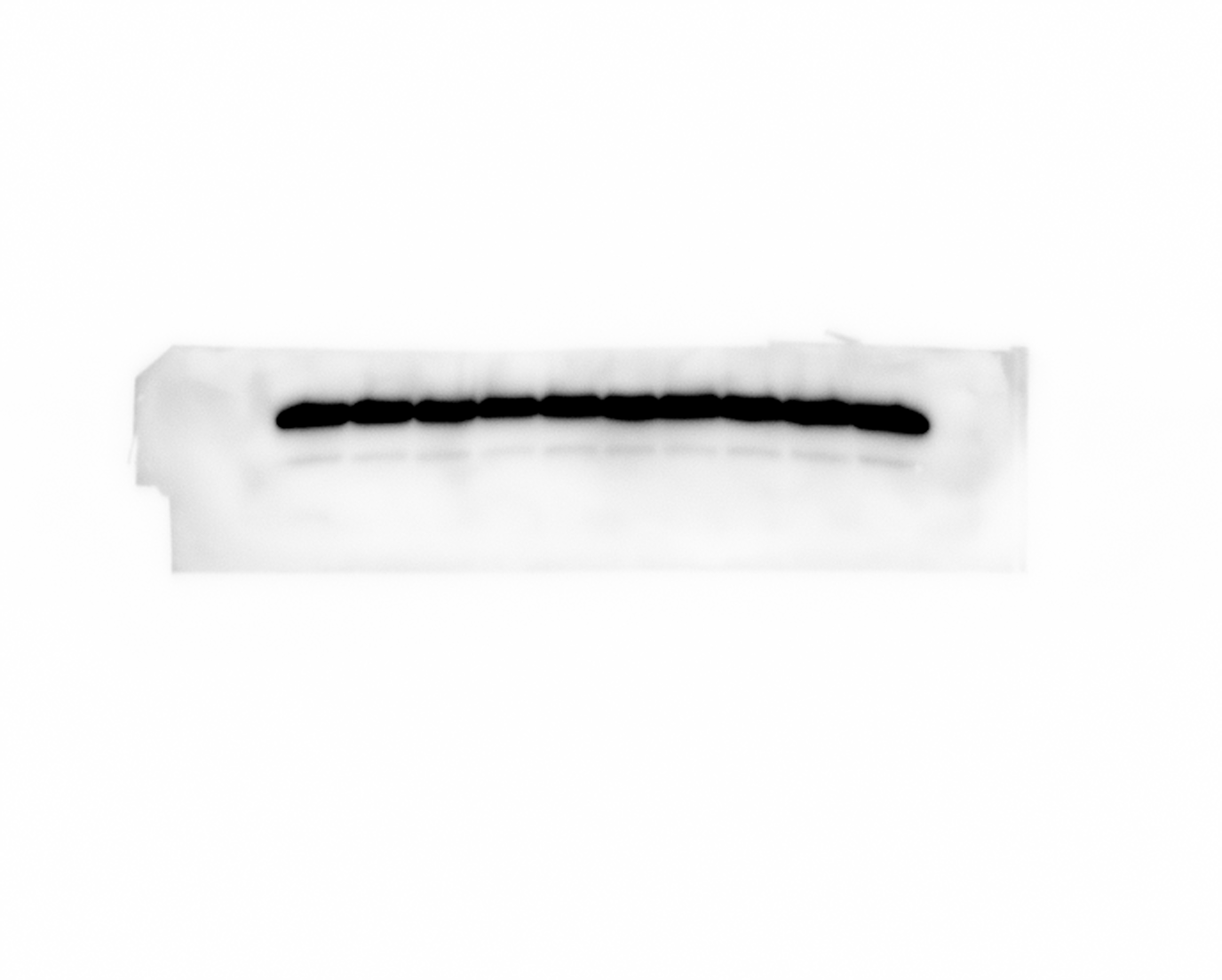

Supplement: Supplementary file 4 [file Image9.TIF]

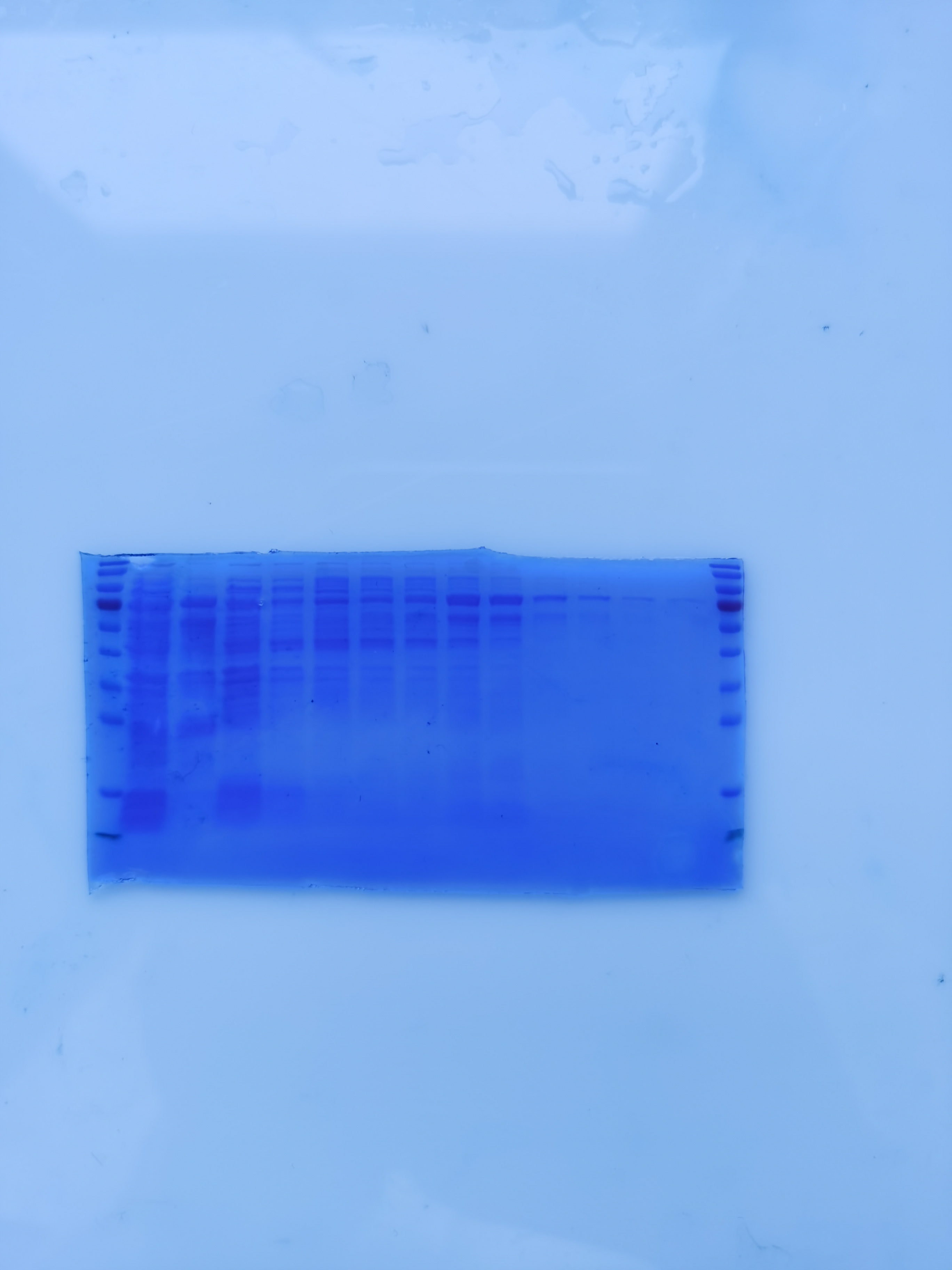

Supplement: Supplementary file 5 [file Image1.JPEG]

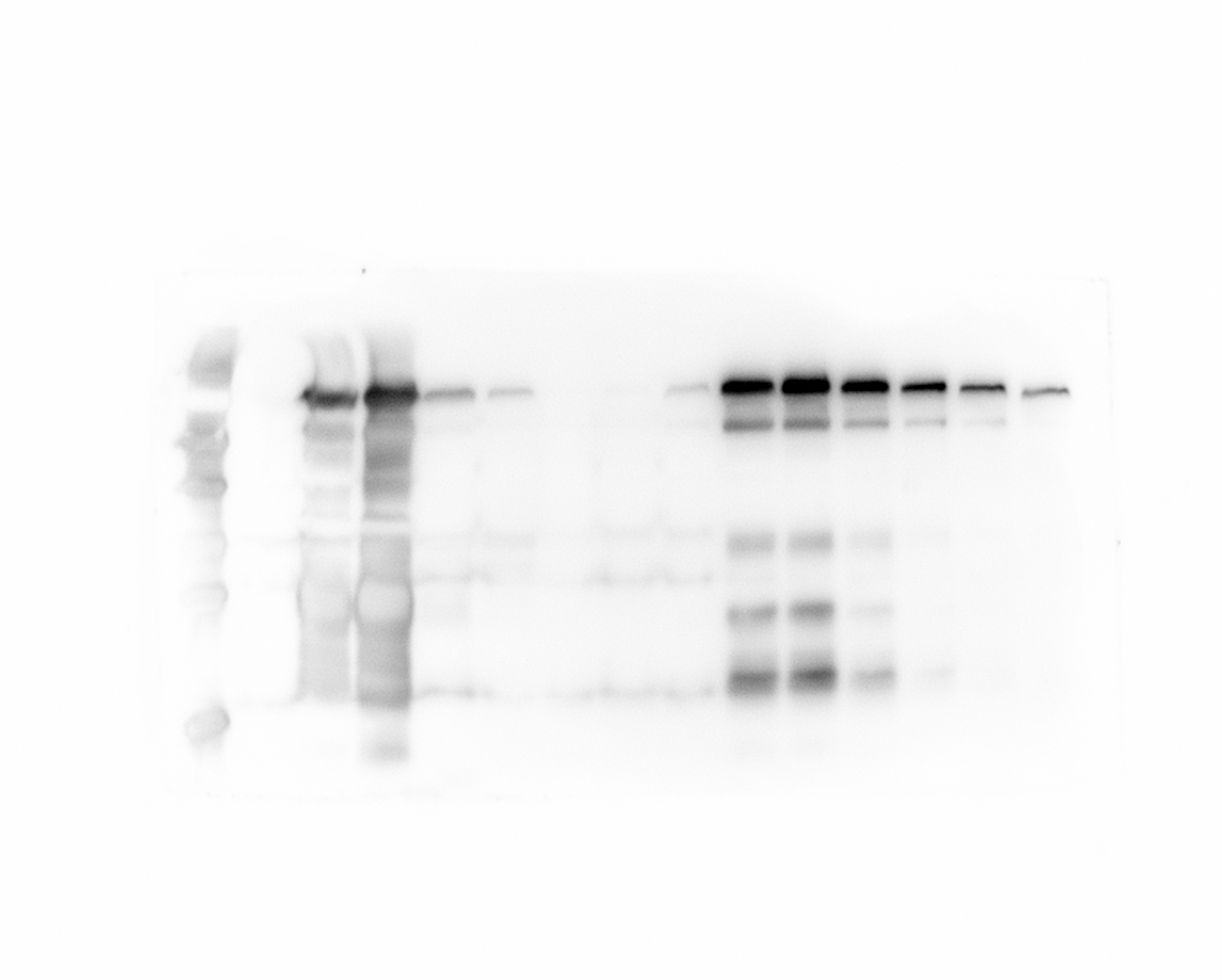

Supplement: Supplementary file 6 [file Image2.TIF]

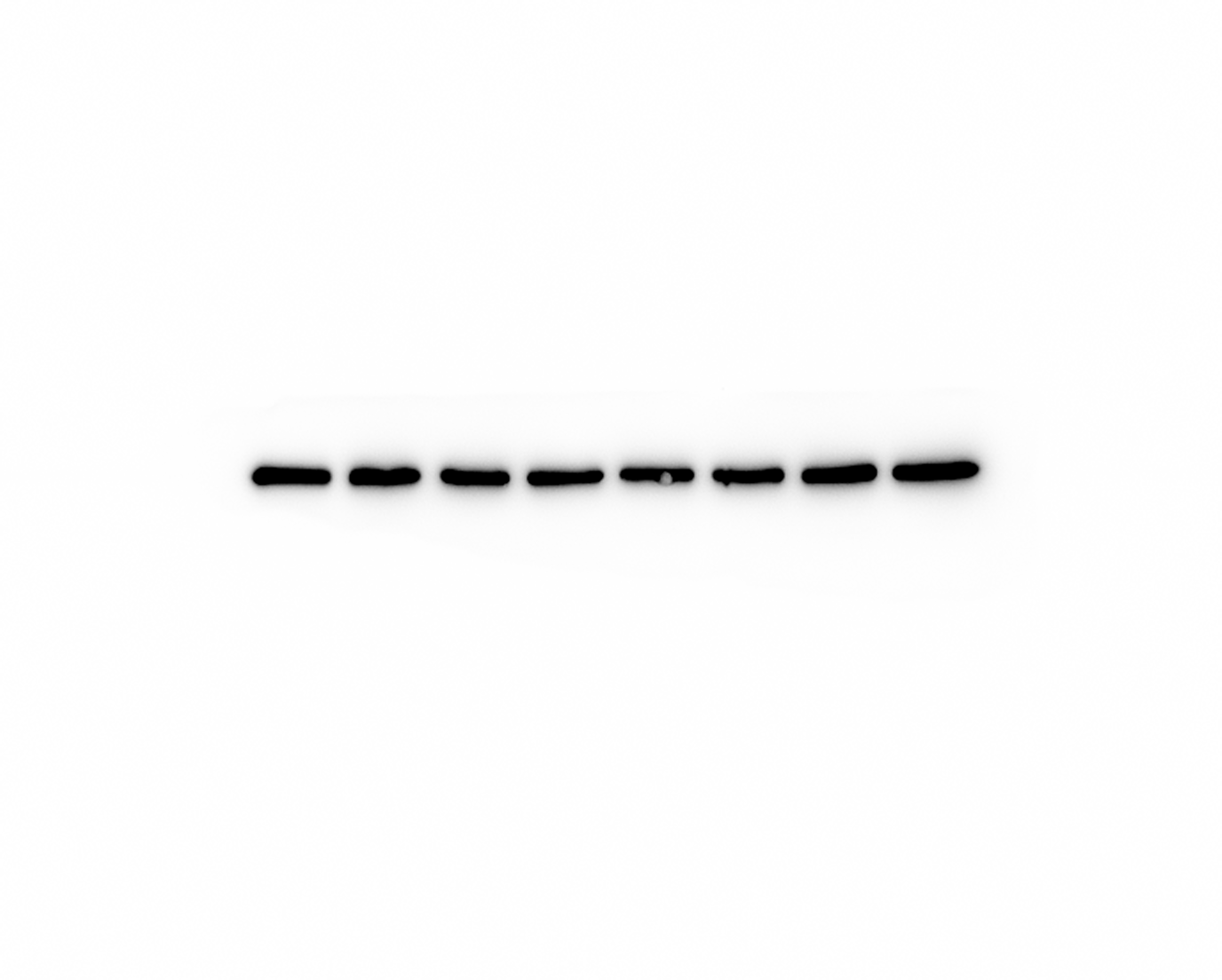

Supplement: Supplementary file 7 [file Image11.TIF]

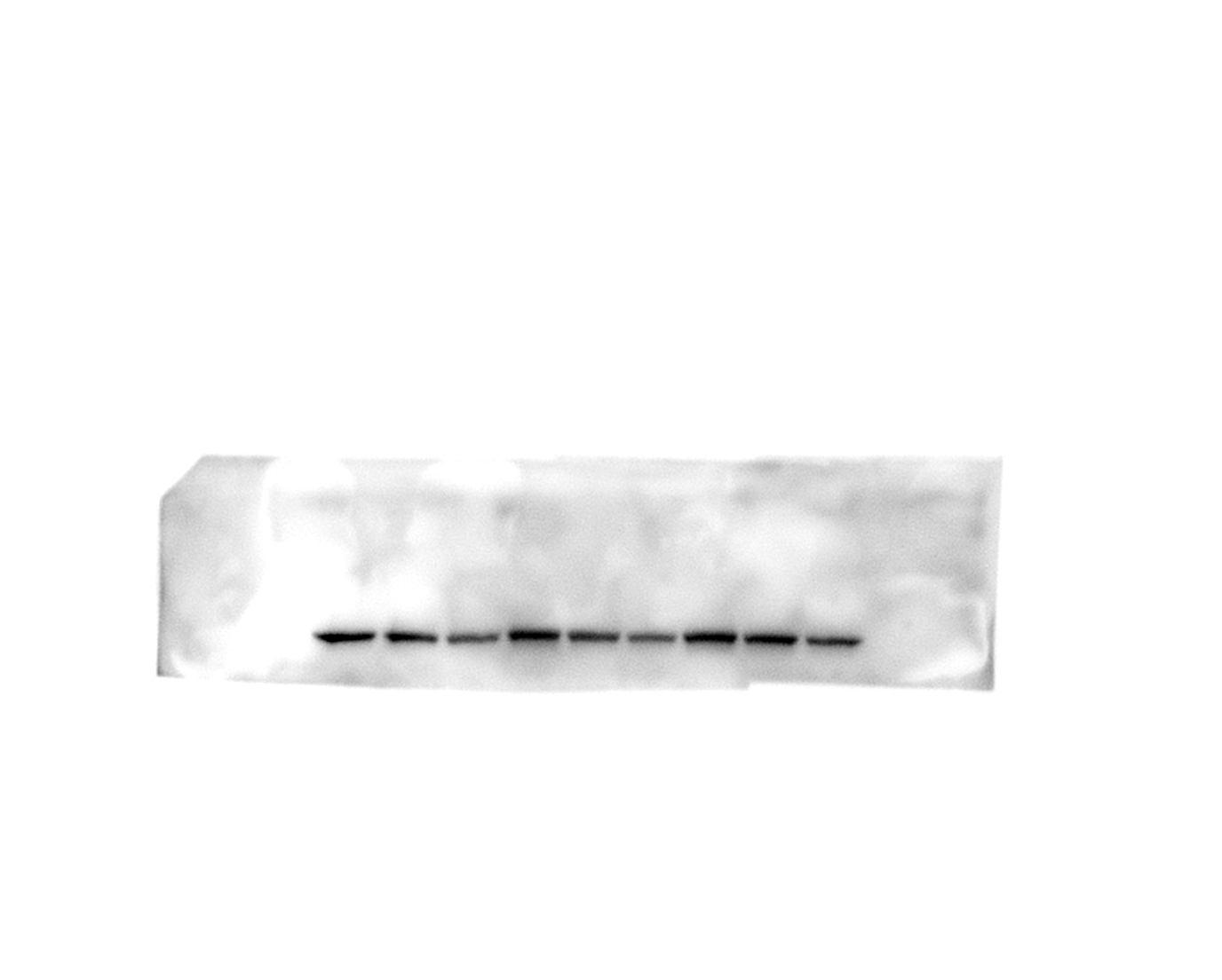

Supplement: Supplementary file 8 [file Image10.TIF]

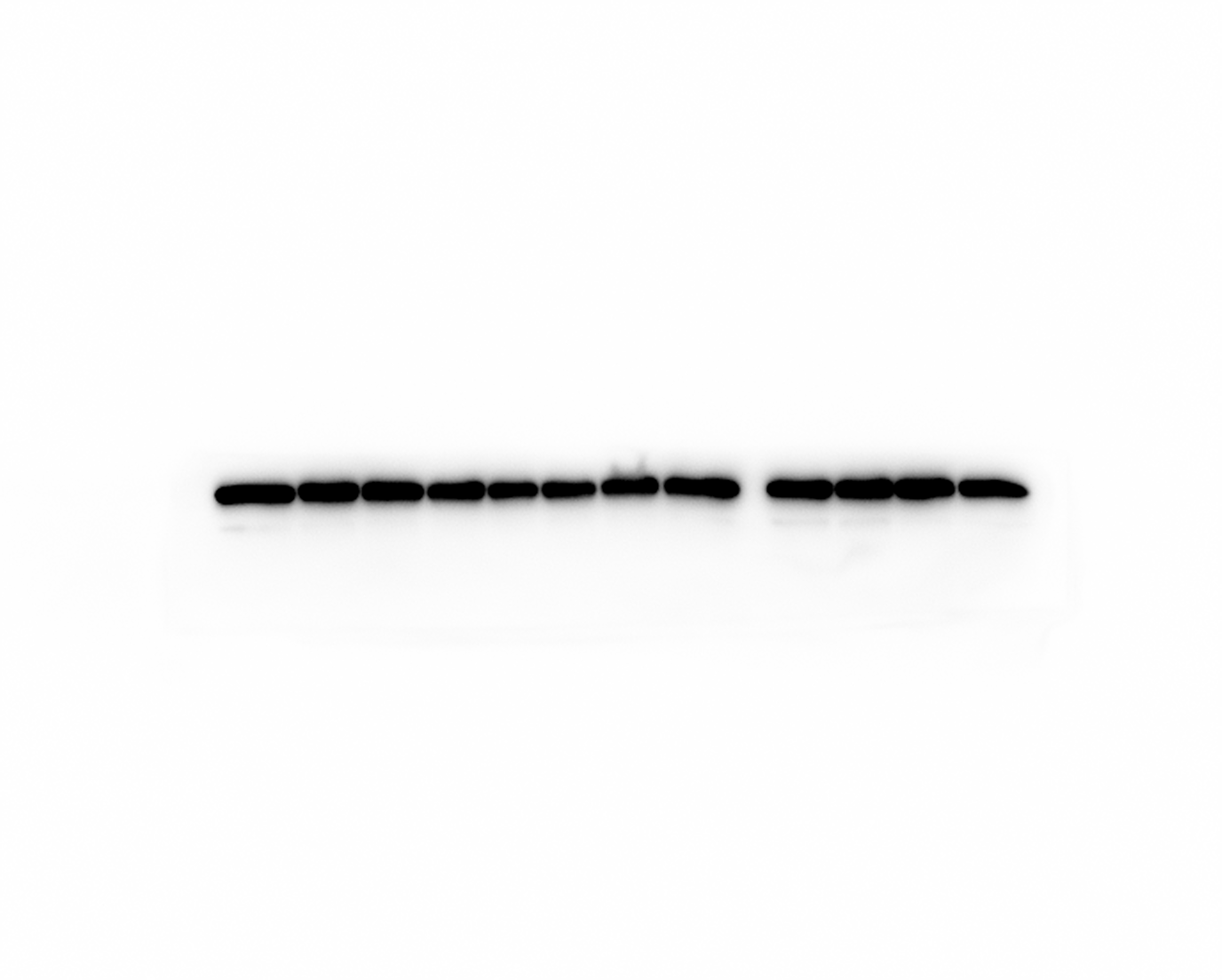

Supplement: Supplementary file 9 [file Image7.TIF]

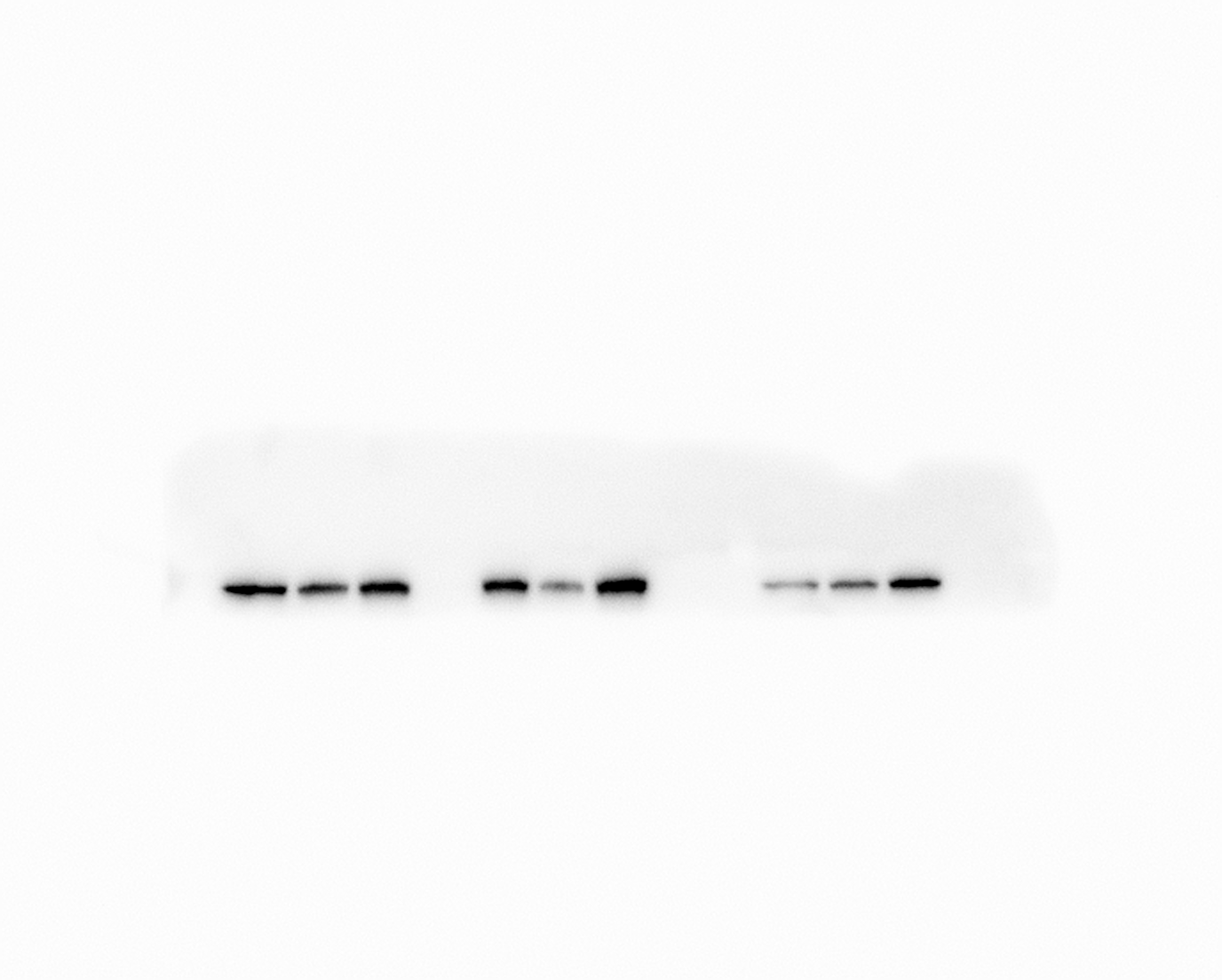

Supplement: Supplementary file 10 [file Image8.TIF]

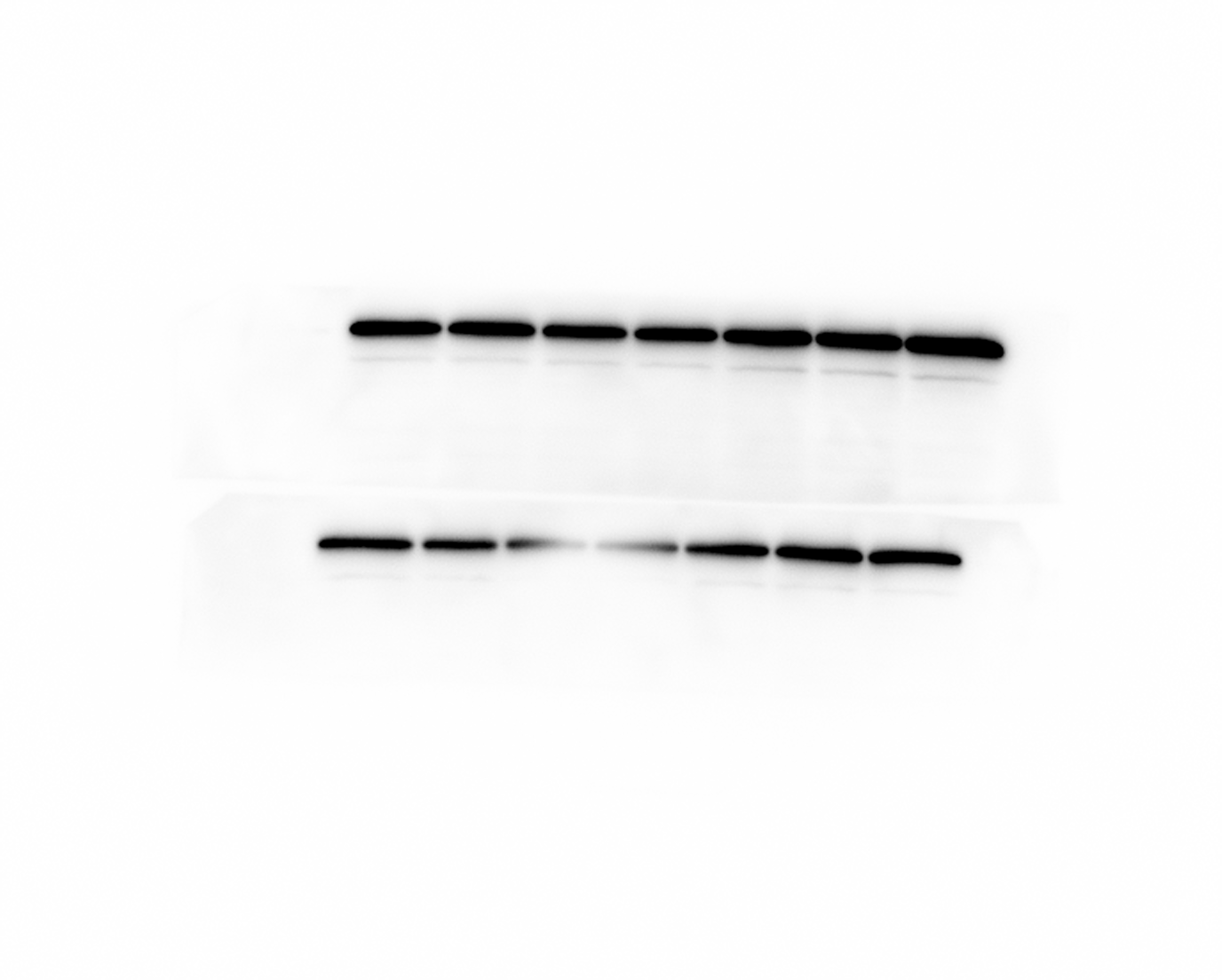

Supplement: Supplementary file 11 [file Image5.TIF]

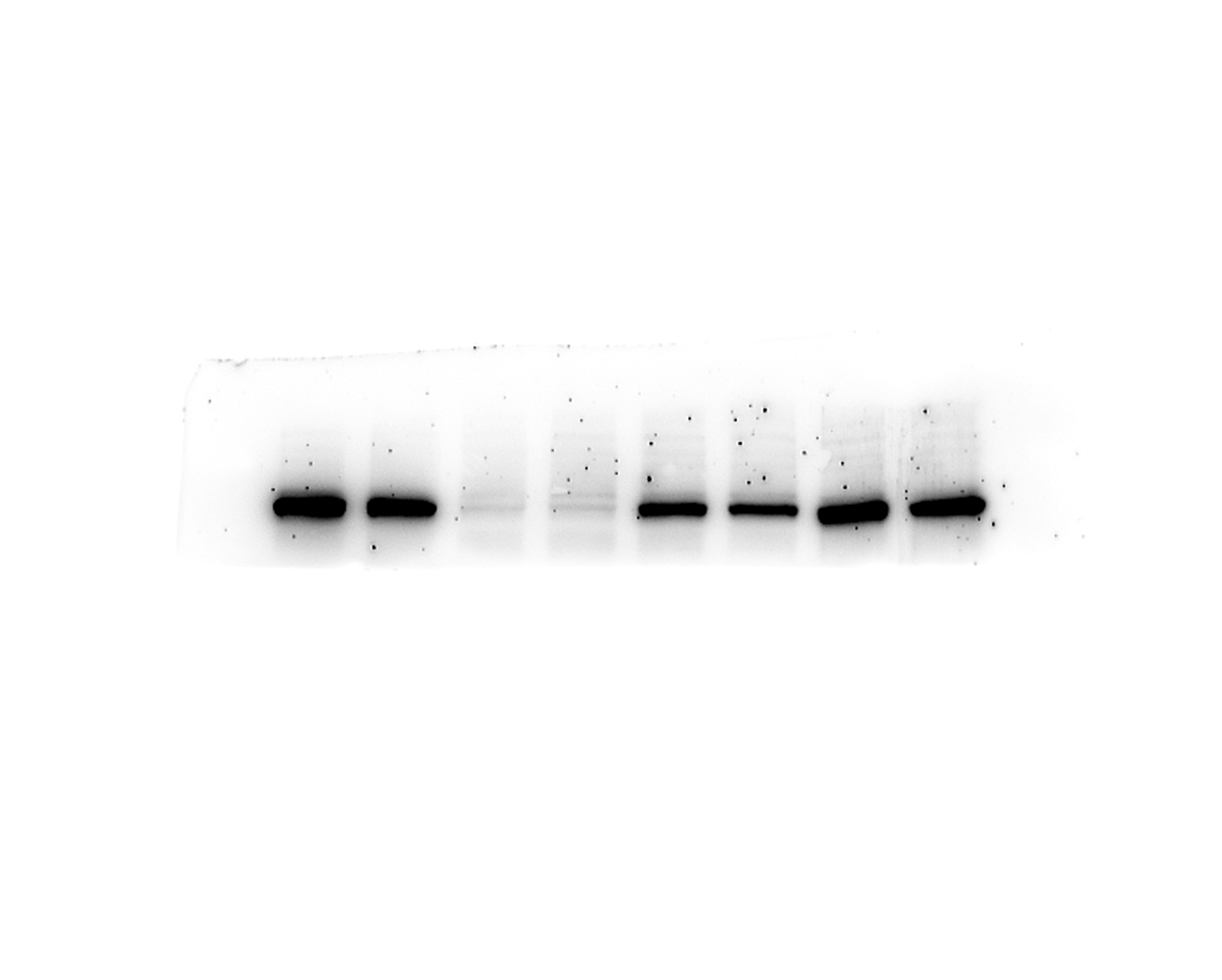

Supplement: Supplementary file 12 [file Image12.TIF]
